# Rotavirus Viroplasm Fusion and Perinuclear Localization Are Dynamic Processes Requiring Stabilized Microtubules

**DOI:** 10.1371/journal.pone.0047947

**Published:** 2012-10-23

**Authors:** Catherine Eichwald, Francesca Arnoldi, Andrea S. Laimbacher, Elisabeth M. Schraner, Cornel Fraefel, Peter Wild, Oscar R. Burrone, Mathias Ackermann

**Affiliations:** 1 Institute of Virology, University of Zurich, Zurich, Switzerland; 2 Institute of Veterinary Anatomy, University of Zurich, Zurich, Switzerland; 3 International Centre for Genetic Engineering and Biotechnology, Trieste, Italy; 4 Dipartimento Clinico di Scienze Mediche, Chirurgiche e della Salute, Università degli Studi di Trieste, Trieste, Italy; Tulane University, United States of America

## Abstract

Rotavirus viroplasms are cytosolic, electron-dense inclusions corresponding to the viral machinery of replication responsible for viral template transcription, dsRNA genome segments replication and assembly of new viral cores. We have previously observed that, over time, those viroplasms increase in size and decrease in number. Therefore, we hypothesized that this process was dependent on the cellular microtubular network and its associated dynamic components. Here, we present evidence demonstrating that viroplasms are dynamic structures, which, in the course of an ongoing infection, move towards the perinuclear region of the cell, where they fuse among each other, thereby gaining considerably in size and, simultaneouly, explaining the decrease in numbers. On the viral side, this process seems to depend on VP2 for movement and on NSP2 for fusion. On the cellular side, both the temporal transition and the maintenance of the viroplasms are dependent on the microtubular network, its stabilization by acetylation, and, surprisingly, on a kinesin motor of the kinesin-5 family, Eg5. Thus, we provide for the first time deeper insights into the dynamics of rotavirus replication, which can explain the behavior of viroplasms in the infected cell.

## Introduction

Rotavirus, a member of the *Sedovirinae* subfamily within the *reoviridae* family, is an icosahedral, non-enveloped, triple-layered particle that encapsidates a genome consisting of eleven segments of double-stranded RNA (dsRNA). During the entry into the host cell, the VP4-VP7 outer layer is lost, yielding a double-layered particle (DLP). The DLP becomes transcriptionally active once released into the cytoplasm, producing eleven species of viral positive single-stranded RNA (+ssRNA) [1], [2], [3], [4], [5], [6], [7], [8]. Each +ssRNA codes for one protein, with the exception of the genome segment 11 that encodes for NSP5 and, depending on the viral strain, for NSP6, making a total of twelve proteins, six structural proteins (VP1, VP2, VP3, VP4, VP6 and VP7) and six non-structural proteins (NSP1, NSP2, NSP3, NSP4, NSP5 and NSP6) [9]. The viral primary translation is required: i) for the subversion of the host translation machinery, mediated by NSP3 [10], [11], [12], [13], [14]; ii) to antagonize the host innate immune response, mediated by NSP1 [15] and iii) for the formation of viroplasms, the cytosolic machinery of replication [16], [17], [18], [19], [20], [21]. These structures correspond to electron-dense inclusion bodies without lipidic membranes surrounded by polyribosomes [16]. Four structural proteins, VP1, VP2, VP3 and VP6 and three non-structural proteins, NSP2, NSP5 and NSP6, as well as nucleic acids (+ssRNA and dsRNA) have been identified to make part of the viroplasm [16]. Discerning the distribution of these viral proteins in and around the viroplasms was possible using monospecific antisera. In this manner, it is possible to subdivide the viroplasm in an interior and an exterior domain, based on their antibody-accessible protein content. Thus, the interior domain, as determined by fluorescence-staining studies is constituted mainly by NSP2, NSP5, the core components (VP1, VP2, VP3) and +ssRNA and dsRNA and, as denoted by electron microscopy, correspond to electron dense inclusions [22]. The interior domain is believed to be involved in the transcription of a +ssRNA template, replication of the dsRNA genome segments and in the packaging of the genomic segments into the newly assembled viral cores. The newly assembled cores are directed to an exterior domain that is characterized by the presence of VP6. Additionally, the viroplasms are surrounded by a region rich in VP7, as detected by immunofluorescence, that corresponds to ER or assembled TLPs (triple layered particles) within ER [21], [23].

The viral proteins NSP5, VP2 and NSP2 are directly involved in the formation of viroplasms and act as recruiters for the components of the viral replication intermediates. Following co-expression of NSP5 with NSP2 or VP2, and in the absence of other viral proteins, cytosolic viroplasm-like structures (VLS) are formed, that have been termed VLS(NSP2)i or VLS(VP2)i when induced by co-expression of NSP5 with either NSP2 or VP2, respectively [17], [19]. These structures are morphologically almost identical to rotavirus viroplasms. However, VLSs represent a very useful tool, as a simplified model to study viroplasms within a host in an *in-vivo* approach.

After 3 to 4 hours post-infection (hpi), it is possible to distinguish numerous small punctuated viroplasms in the cytoplasm of infected cells that can reach up to 10–20 µm in size at late stages of infection. In a previous study, we have shown viroplasm formation from early to late times post-infection using a stable-transfected MA104 cell line expressing NSP2-EGFP (NSP2-EGFP/MA104) infected with rotavirus and suggested that the temporal transition in number and size of viroplasms could be the result of a process of fusion of small viroplasms concomitant with an increase in the protein content of the viroplasm components [18], [21], [22], [23].

Microtubules (MTs), the largest cytoskeletal components, are involved in intracellular transport, organelle positioning, cell shape and motility. More complex MT-structures are involved in the formation of centrioles and axonemes and in cell division, like the formation of mitotic spindles and midbody. A wide range of post-translational modifications (PTMs), unique for tubulin or shared with other proteins, generate chemical differences that are sufficient to confer specific cellular functions to MTs. Specific PTMs for MTs are detyrosination, Δ2-tubulin generation, polyglutamylation, polyglycinilation and acetylation [24]. All these PTMs, with the exception of acetylation, occur at the C-terminal region of α/β-tubulin. Acetylation modifies lysine 40 at the N-terminal region of α-tubulin, and is the most frequent PTM associated with MT-stabilization [25], [26]. MTs dynamics and function are modulated by interactions with other proteins, molecular motors and non-motor microtubule-associated proteins (MAPs). Up to date, two major families of molecular motors, dyneins and kinesins are known to generate the force, upon interaction with MTs, required for various intracellular functions including intracellular transport. As molecular motors, these enzymes convert the chemical energy of ATP hydrolysis into mechanical energy and force production [27]. MAPs are a heterogeneous group that includes stabilizing-MT proteins, such as tau, MAP1 and MAP2, as well as destabilizing proteins like spatin and katanin, and the MT plus-end tracking proteins (+TIPs) [28]. Despite the importance of the MT-network in many cellular processes, its role in viroplasms dynamics, has been poorly addressed [29], [30], [31]. The lack of a robust reverse genetic system for rotavirus [32], [33], [34] as well as a method for viroplasms purification, hamper the study of viroplasms formation, dynamics, composition or interaction with host components. In this report, we address some fundamental questions on the rotavirus life cycle using alternative methodologies. We present evidence for viroplasm-viroplasm fusion and perinuclear condensation, demonstrating that they are dynamic structures. We show that both the temporal transition as well as the maintenance of viroplasms require the MT-network and a kinesin motor from the Eg5 family. Additionally, in a simplified model for viroplasm interaction with MT-network, using VLS we show that NSP2 is necessary for viroplasm fusion while VP2 is necessary for their perinuclear localization.

## Materials and Methods

### Cells and Viruses

MA104 (embryonic rhesus monkey kidney, ATCC® CRL-2378) and CV-1 (african green monkey kidney fibroblasts, generously donated by Max L. Nibert, Harvard Medical School [35]) cells were cultured in Dulbecco’s modified Eagle’s media (DMEM, Gibco, BRL) supplemented with 10% fetal bovine serum (FBS, BRAND). NSP5-EGFP/− and NSP2-EGFP/MA104 cell lines [18] were cultured in DMEM supplemented with 10% FBS and 800 µg/ml geneticin. Vero 2.2 cells [36] were cultured in DMEM supplemented with 10% FCS and 500 µg/ml geneticin. Simian rotavirus strain SA11 (G3, P6 [1]) and porcine rotavirus strain OSU (G5, P9 [7]) were propagated and grown as described by Estes et al., 1979 [37].

### Antibodies and Reagents

Guinea pig polyclonal antisera specific for NSP5 and VP2 were used as described previously [18], [19]. Mouse monoclonal antibody (mAb) anti-VP6 (clone 2F) was a gift from Dr. N. Mattion (CEVAN, Buenos Aires, Argentina). Mouse mAb anti-alpha-tubulin (clone B-5-1–12); Mouse mAb anti-alpha tubulin acetylated (clone 6–11B-1), mouse mAb anti-GAPDH (clone GAPDH-71.1) and rabbit polyclonal anti-actin were obtained from Sigma-Aldrich. Rabbit polyclonal anti-vimentin (H-89) was obtained from Santa Cruz Biotechnology, USA. Goat anti-mouse immunoglobulin G (IgG) (H+L) Alexa 488; rabbit anti-mouse F (ab’)2 fragments Alexa 594; goat anti-mouse IgG Alexa 647 and goat anti-guinea pig IgG(H+L) Alexa 488 were obtained from Molecular Probes, Invitrogen, USA. Goat polyclonal anti-guinea pig IgG conjugated to rhodamine was obtained from KPL, USA. Goat polyclonal anti-mouse IgG (Fab)’-peroxidase was obtained from Sigma-Aldrich and rabbit polyclonal anti-guinea pig Ig-peroxidase was obtained from DakoCytomation, Denmark. Mouse mAb anti alpha-tubulin was directly conjugated to Atto 488 using lightning-link™ Atto 488 conjugation kit from Innova Bioscience, UK. Nocodazole, vinblastine, Paclitaxel (taxol) from *Taxus brevifolia* and monastrol were purchased from Sigma-Aldrich.

### Plasmid Constructions

Plasmid pHSV[NSP2-mCherry] was obtained by PCR amplification of the NSP2-mCherry fragment from pCI-NSP2-mCherry using specific primers to containing flanking SalI and EcoRI restriction sites, followed by ligation between the SalI and EcoRi sites of pHSVs [38]. Plasmid pCI-NSP2-mCherry was obtained by PCR amplifications of NSP2 strain SA11, using specific primers to incorporate EcoRI and MluI at 5′and 3′ ends and mCherry, from pRSET-mCherry [39], using specific primers to incorporate SalI and XmaI at 5′ and 3′ ends. Both fragments, NSP2 and mCherry, were in frame-ligated into pCI-Neo (Promega). Plasmid pHSV-1[mRFP-Δ92VP2] construction was described by Laimbacher et al., 2012 [40] and HSV-1 bacterial artificial chromosome fHSVΔpacΔ27 and plasmid pEBICP27 were described by Saeki et al., 2001 [41]. All oligonucleotides were obtained from Microsynth AG, Switzerland and described in Table S1.

### Rotavirus Titration

Virus was activated with 50 µg/ml trypsin for 30 min at 37°C and serially diluted from 1×10^−2^ to 10^−7^ in DMEM. NSP5-EGFP/MA104 cells, seeded in 24-wells plates with coverslips, were infected with 25 µl of each viral dilution. After 1 h of adsorption at 4°C, virus was removed and cells were incubated at 37°C. At 4 hours post-infection (hpi), cells were fixed with 2% paraformaldehyde (PFA) in phosphate buffer saline (PBS) [137 mM NaCl; 2.7 mM KCl; 8.1 mM Na_2_HPO_4_ and 1.74 mM KH_2_PO_4_ pH7.5] for 10 min at room temperature. Nuclei were stained with a 70 nM 4,6-diamino-2-phenylindole (DAPI) solution and coverslip were mounted in slides with prolong Gold (Molecular Probes, Invitrogen). Samples were observed using a fluorescence microscope with a 40X lens. A cell containing viroplasms was considered as 1 viroplasm-forming unit (VFU). The average of cells with viroplasms of three quantified fields per each virus dilution was determined and then, estimated the total number of cells containing viroplasms in the whole preparation. The virus titer was estimated as [total number of cell with viroplasms×virus dilution/volume of virus added in mililiters]. It was found that a multiplicity of infection (MOI) of 25 VFU per cell is required to infect all cells in a monolayer.

### Live Imaging Acquisition of Viroplasms

NSP5-EGFP/or NSP2-EGFP/MA104 cell lines were seeded into 35 mm glass bottom dishes (glass N°1, 14 mm diameter, Mat Tek, corporation, USA) and infected with simian rotavirus SA11 [MOI; 25 VFU/cell]. At 4 hpi, cells were placed into a chamber on a Zeiss LSM510 META Axiovert 200 M reverse microscope at 37°C in a 5% CO_2_ atmosphere. Transmitted light images were acquired with a 63×objective and a 3CCD camera. Image acquisition was performed with intervals of 20 min. Frames were analyzed using Image J 1.42 q software (W.Rasband/NIH, USA). Images were prepared for publication using Photoshop CS (Adobe) and PowerPoint (Microsoft) software.

### Transmission Electron Microscopy

MA104 cells were seeded at 8×10^4^ cells in a 2 cm^2^ well onto sapphire discs and infected with simian rotavirus SA11 [MOI; 250 VFU/cell]. For the high pressure freezing procedure, cells were pre-fixed in 0.25% glutaraldehyde (GA) and put under high pressure freezing (HPM010, BAL-TEC) [42]. The frozen cells were transferred into a freeze substitution unit (FS 7500, Boeckeler Instruments, Tucson, AZ, USA) precooled at −88°C for substitution with acetone and subsequent fixation with 0.25% GA and 0.5% osmium tetroxide rising the temperatures gradually to +2°C [43]. Then, samples were embedded in epon at 4°C followed by polymerization at 60°C for 2.5 days Alternatively, cells were fixed with 2.5% GA in 100 mM Na/K-phosphate buffer, pH 7.4 for 1 h at 4°C and kept into 100 mM Na/K-phosphate buffer overnight at 4°C. Afterwards, samples were post-fixed in 1% osmium tetroxide in 100 mM Na/K-phosphate buffer for 1 h at 4°C, dehydrated in a graded ethanol serie starting at 70% followed by two changes in acetone and embedded in epon. Ultrathin sections (60–80 nm) of cryofixed and conventionally fixed samples were cut and stained with uranyl-acetate and lead citrate before analysis in a transmission electron microscope (CM12, Philips, Eindhoven, The Netherlands) equipped with a CCD camera (Ultrascan 1000, Gatan, Pleasanton, CA, USA) at an acceleration of 100 kV.

### Immunofluorescence

For cytoskeleton detection (microtubules, actin and intermediate filaments), cells were fixed in cold methanol for 3 min at −20°C. Alternatively, cells were fixed with 2% PFA for 10 min at room temperature. Coverslips were permeabilized for 5 min in PBS containing 0.1% Triton X-100, and blocked in PBS containing 1% bovine serum albumin (BSA) for 30 min. Primary and secondary antibodies were diluted in PBS containing 1% BSA and incubated for 45 min, all at room temperature in a humid chamber. Nuclei were stained with DAPI. Images were acquired using a CLSM (Leica, DM 5500 Q) equipped with a 63×1.3 oil objective. Data was analyzed with Leica Application Suite (Mannheim, Germany) and the Imaris software package (Bitplane, Switzerland). Images were prepared for publication using PowerPoint (Microsoft) software.

### Viroplasms Perinuclear Assay

Cells monolayers were infected with rotavirus at MOI of 10 VFU/cell, adsorbed for 1 h at 4°C, and then incubated at 37°C and 5% CO_2_. When indicated, media was removed and drug dissolved in 2% dimethyl sulfoxide (DMSO) in fresh medium, was added. At the selected time points, Image-IT™ LIVE plasma membrane and nuclear labeling kit (Molecular Probes, Invitrogen) was used to quantify the perinuclear condensation of viroplasms. Briefly, cells were fixed with 4% PFA for 15 min at 37°C and incubated for 10 min at room temperature with 5 µg/ml WGA conjugated to Alexa 594 and 2 µM Hoescht 33342 (Molecular Probes, Invitrogen). Subsequently, cells were permeabilized with 0.01% Triton X-100 in PBS for 5 min at room temperature and incubated with a guinea pig anti-NSP5 antibody followed by a goat anti-guinea pig conjugated to Alexa 488 secondary antibody (Invitrogen). Coverslips were mounted using Prolong Gold (Molecular Probes, Invitrogen). Images were acquired using a CLSM (Leica, DM 5500 Q) equipped with a 63×1.3 oil objective. Data were analyzed with Leica Application Suite (Mannheim, Germany) and the Imaris software package (Bitplane, Switzerland). The total area of the cells (c), the area of distribution of viroplasms (v) and nuclei (n) were determined using ImageJ 1.42 q (W. Rasband, NIH, USA). The condensation of the viroplasms to the perinuclear space was expressed as [V/C] ratio, where V = v−n and C = c–n. Values and statitical analysis was performed with Microsoft® Excel 2008 for MAC, version 12.3.1, using a two-tailed paired Student’s t-test.

### Determination of Numbers, Areas and Frequencies of Viroplasms

The viroplasms area and numbers were obtained using the ‘analyze particles tool’ from ImageJ 1.42 q (W. Rasband, NIH, USA). Values, frequencies and statistical analysis, were perfomed with Microsoft® Excel 2008 for MAC, version 12.3.1, two tail-paired Student’s t-test.

### Immunoblotting

In general, cells were seeded in 12-well plates, lysed at the indicated time points in 30 µl lysis buffer and further processed as described by Eichwald et al., 2004 [44].

### VLS (VP2i) and (NSP2i)

VP2 and NSP2 were expressed using helper virus-free HSV-1 amplicon vectors [45]. NSP5-EGFP/MA104 cells were infected with 1 TU (transducing unit)/cell of pHSV [NSP2-mCherry] or pHSV [mRFP-Δ92VP2] in DMEM supplemented with 2% FCS. At 23 hpi, the medium was replaced with medium containing 10 µM nocodazole or monastrol and the cultures were kept for 1 h at 37°C. Finally, the cells were treated for immunostained and images were acquired by CLSM as described above. VLS perinuclear localization value was determined as [VA-N]/VA ratio, where VA is the area occupied by VLS including the nucleus and N is the nuclear area.

## Results

### Viroplasms Increase in Size, Decrease in Number and Move Towards the Perinuclear Region during Viral Infection

**Figure 1:**
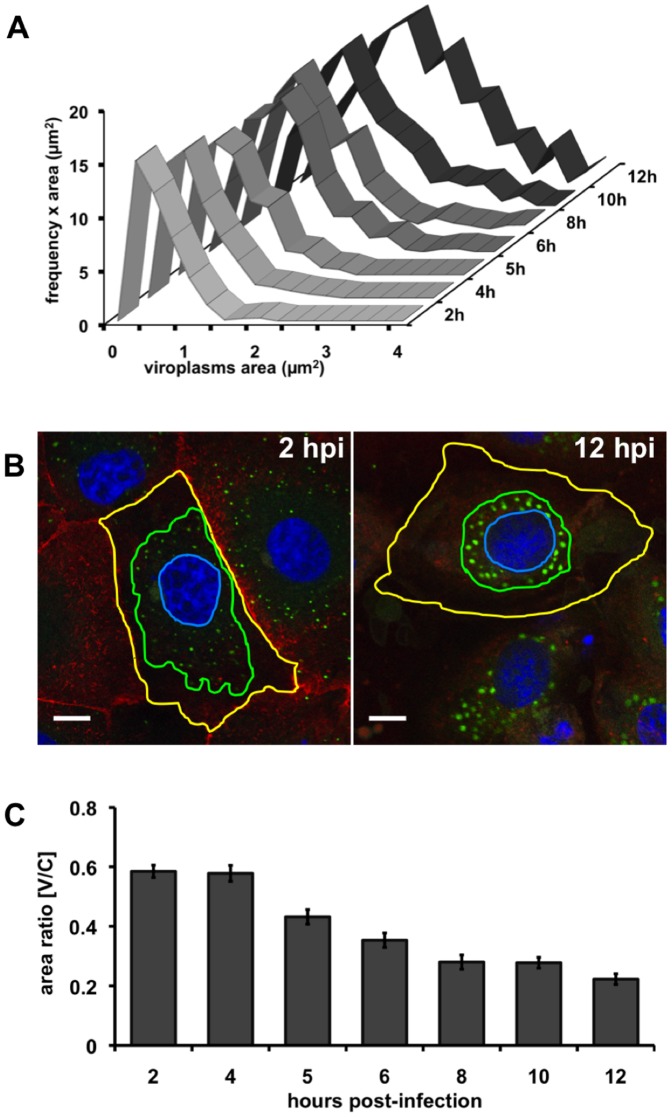
Viroplasms are dynamic entities. Viroplasms area and distribution on cell was determined on SA11-infected NSP5-EGFP/MA104 cells. (**A**) The area of individual viroplasms was plotted against the frequencies of the individual size categories at different times post-infection. The ‘Y’ axis corresponds to the number of viroplasms found on defined dimension windows (frequency), multiplied by the maximum value of the window (area) (**B**) Cell staining for viroplasms perinuclear condensation assay; viroplasms were detected with NSP5-EGFP (green), plasma membrane was stained with WGA-Alexa 594 (red) and nuclei were stained with Hoechst 33342 (blue). Yellow, green and blue lines represent the total area of the cell, the area of the cell where viroplasms are distributed and the nuclei, respectively. Scale bar is 10 µm. (**C**) Plot for perinuclear condensation [V/C ratio] determined at various times post-infection. Data is presented as means ± SEM, n>50 cells.

In order to visualize the number, size and location of viroplasms throughout the time course of infection, we made use of the stable transfected MA104 cells expressing NSP5-EGFP (NSP5-EGFP/MA104) [18], [19], [46], [47] infected with simian rotavirus SA11 (Figure S1). Comparable results were obtained when analyzing the size of the viroplasms (measuring the area) and plotting the distribution of size frequency at different times post-infection (Figure 1A). With this kind of analysis, it was possible to establish a gradual transition in the size of viroplasms from small at early times (2 to 5 hpi) to large at late times (6 to 12 hpi) post-infection. In addition, small viroplasms (ranging from 0.025–1.5 µm^2^) were prevalent during the whole course of infection, while larger ones (ranging from 1.5 up to >4 µm^2^) were established from around 6 hpi. By considering the frequency rather than the average size, it was possible to reveal the appearance of large viroplasms. Staining of the plasma membrane and the nucleus of NSP5-EGFP/MA104 cells infected with simian rotavirus SA11 (Figure 1B) supported detection of a second event consisting of a gradual movement of viroplasms to the perinuclear region during the infection period. This was quantified by measuring the area of the cell containing viroplasms (V) relative to the total area of the cytoplasm (C). As depicted in Figure 1C, the viroplasms transition to the perinuclear area occurred from 5 to 8 hours post-infection.

### Viroplasms Fusion

**Figure 2:**
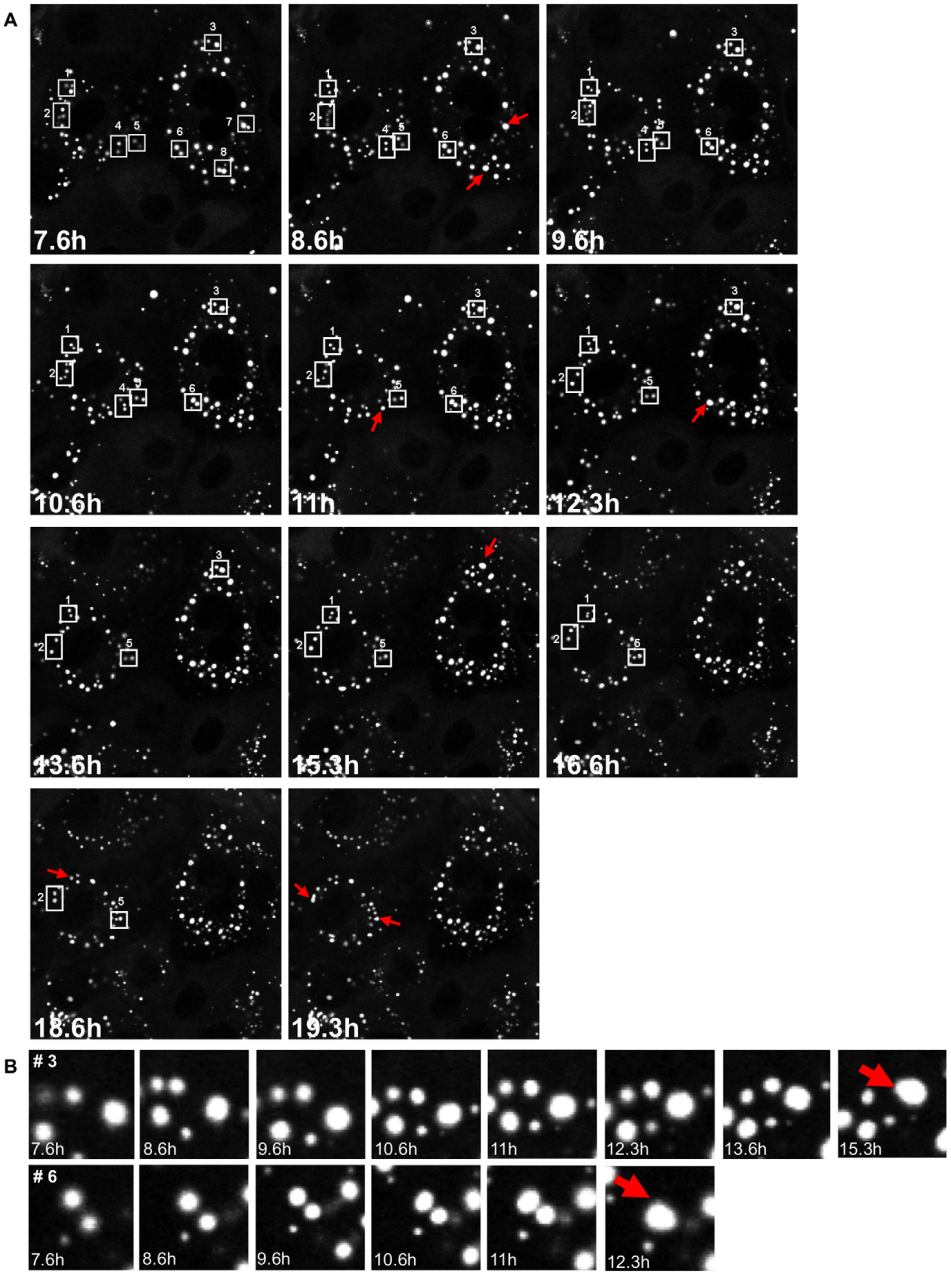
Time-lapse confocal microscopy of SA11-infected NSP5-EGFP/MA104 cells. Acquisition was performed during an infection period from 4 to 22 hpi. (**A**) The most representative frames are shown. Clusters of viroplasms to be fused are indicated in numerated white boxes; a red arrows point to fused viroplasms. (**B**) Enlarged images from fusion of viroplasm clusters occurring in white boxes 3 (upper panel) and 6 (lower panel). Red arrows indicate fused viroplasm.

**Figure 3:**
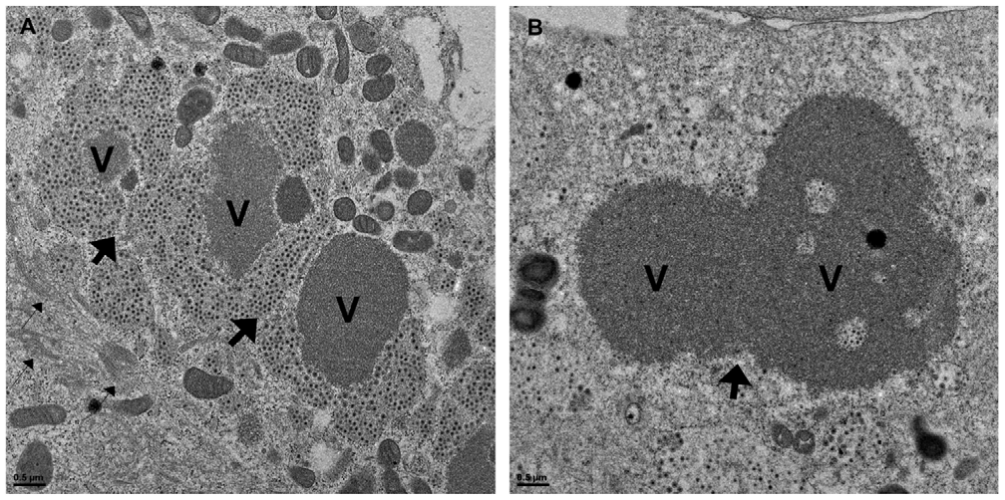
Viroplasms fusion in SA11-infected MA104 cells visualized through high-resolution electron microscopy at 12 hpi. (**A**) The starting process of viroplasms fusion is shown in a group of three viroplasms. (**B**) Viroplasms fused internal domains (V) are evident. Thick and thin black arrows indicate the site of fusion and the presence of MT-bundles, respectively. Scale bars are 0.5 µm.

The change in size of individual viroplasms areas and the perinuclear movement, suggested a dynamic behavior of viroplasms within the cell that could involve active viroplasm-viroplasm fusions. To address this hypothesis, we performed time-lapse confocal microscopy of NSP5-EGFP/MA104 cells infected with simian rotavirus SA11 and followed the fate of individual viroplasms within the cell. To do this, cells were inspected under the microscope from 4 hpi to 20 hpi and images were recorded every 20 min (Video S1). Single frames were analyzed and selected for the identification of viroplasm clusters in the process of fusion. As shown in Figure 2A, up to eight events of fusion could be identified. A series of numerated boxed areas containing individual viroplasms denote, initiation of fusions, while red arrows indicate accomplished fusions. Two such events are depicted with more detail in Figure 2B. The time from the first manifestation of an individual viroplasm to its apparent merge with another one ranged from 4 to 8 hours. Identical results were obtained with NSP2-EGFP/MA104 cells infected with simian rotavirus SA11 (Video S2 and Figures S2A and S2B). At microscopic resolution, it was not possible to discriminate between dense packaging and fusion of viroplasms. However, using high-resolution electron microscopy we observed apparent initial approximation of viroplasms (Figure 3A) and later on, in a more advanced stage, with the electron-dense internal domain seamlessly fused to each other (Figure 3B). This collection of data strongly suggests that the viroplasms are dynamic structures capable of undergoing fusions during the virus replication cycle.

### Viroplasms and MT-network

**Figure 4:**
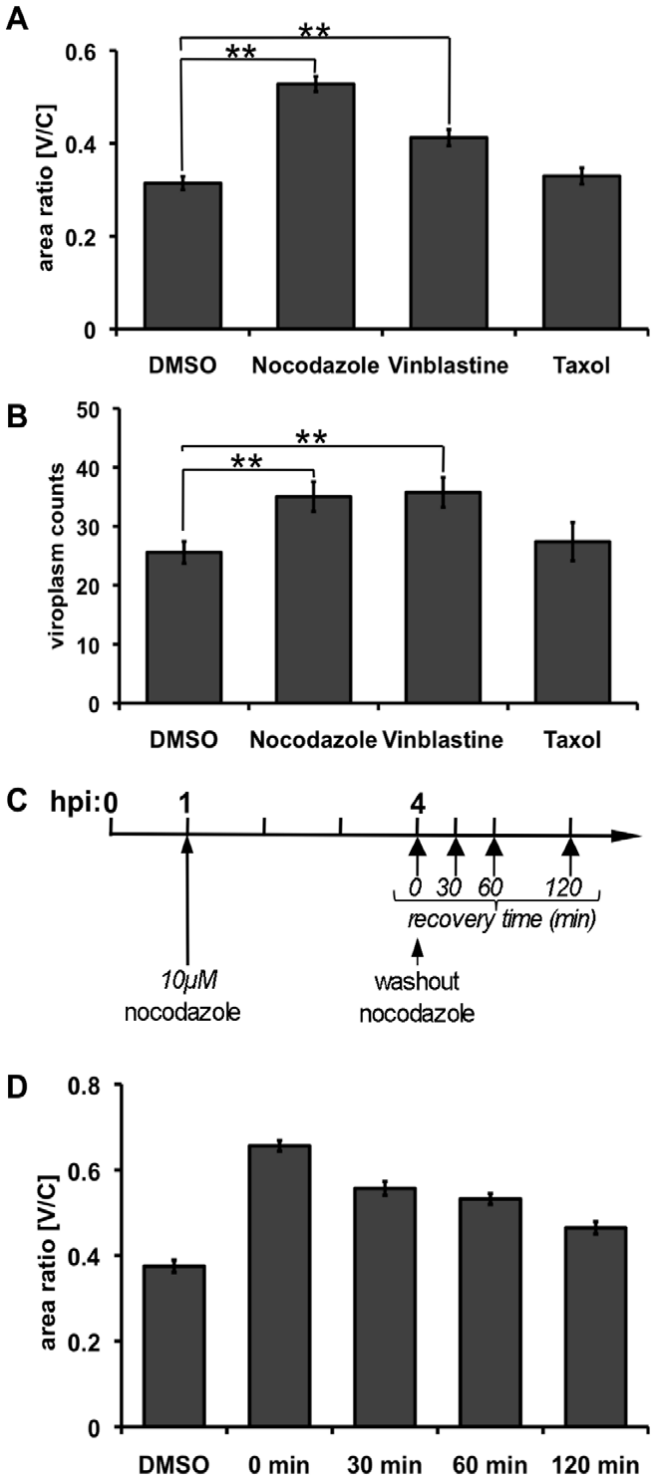
The perinuclear condensation of viroplasms is MT-dependent. SA11-infected CV-1 cells, were treated at 1 hpi with 10 µM nocodazole, vinblastine or taxol and fixed at 6 hpi. Viroplasm perinuclear condensation (**A**) and viroplasms number per cells (**B**) were determined upon each drug treatment. Data is presented as mean ± SEM; t-test, (**) p<0.01, n≥60 cells. (**C**) Experimental-design for MT recovery in rotavirus-infected cells after treatment with nocodazole. (**D**) Viroplasms perinuclear condensation [V/C ratio] was determined for untreated cells at 4 hpi (DMSO) and compared to cells fixed after recovery times of 0, 30, 60 and 120 min after nodocazole removal. Data is presented as mean ± SEM, n>50 cells.

As mentioned above, the cytosolic movements of viroplasms include condensation to the perinuclear area and their fusion. These kinds of movements suggest an active participation of the host's cytosketelon. Indirect immunofluorescence at 6 hpi showed that neither actin- nor intermediate filaments-networks underwent deep changes around rotavirus viroplasms. However, the MT-network showed a tendency to re-distribute around viroplasms as well as to form MT-bundles. Interestingly, we observed that the total distribution of the MT-network in infected-cytosol vary depending of the viral strain (Figure S3). This phenomenon was observed in at least two cell types, CV-1 and MA104. To test the involvement of the MT-network in this process, MT-depolimerizing drugs, such as nocodazole and vinblastine as well as the MT-stabilizing drug taxol were tested in cells infected with simian rotavirus SA11. The ability of viroplasms to move to the perinuclear region was then determined. Our results indicated that nocodazole and vinblastine, compared to mock treatment, significantly inhibited condensation of viroplasms in the perinuclear region. In contrast, the presence of taxol did not significantly affect the same process (Figure 4A). Moreover, the number of individual viroplasms seemed to remain high with nocodazole and vinblastine treatments, while their numbers decreased in either mock- or taxol-treated cells (Figure 4B). Comparable results were obtained for porcine rotavirus strain OSU (Figures S4 B and C), suggesting that these events are not exclusive to one viral strain. To further corroborate that the MT-network is actively involved in the perinuclear re-localization of viroplasms, virus-infected cells were first pulsed with 10 µM nocodazole which was washed out at 4 hpi to allow MTs to recover for 0, 30, 60 and 120 min prior to fixation (Figure 4C). As shown in Figure 4D, viroplasms were able to gradually re-condense in a time dependent manner in the perinuclear region throughout the recovery of the MT-network. These results provide strong evidence that the MT-network is directly involved in the perinuclear re-condensation of viroplasms.

Although viral fitness presents a slightly reduced delay when MTs are depolimerized with nocodazole, no differences were observed in the detection of the viral genome segments by silver staining (Figures S5 A and B). Similar observations concerning viral fitness in nocodazole treated cells have been made also for other reoviridae members, including mammalian orthoreovirus T1L and T3D^N^ treated with nocodazole [35], [48].

### Viroplasms Stabilize the MT-network

**Figure 5:**
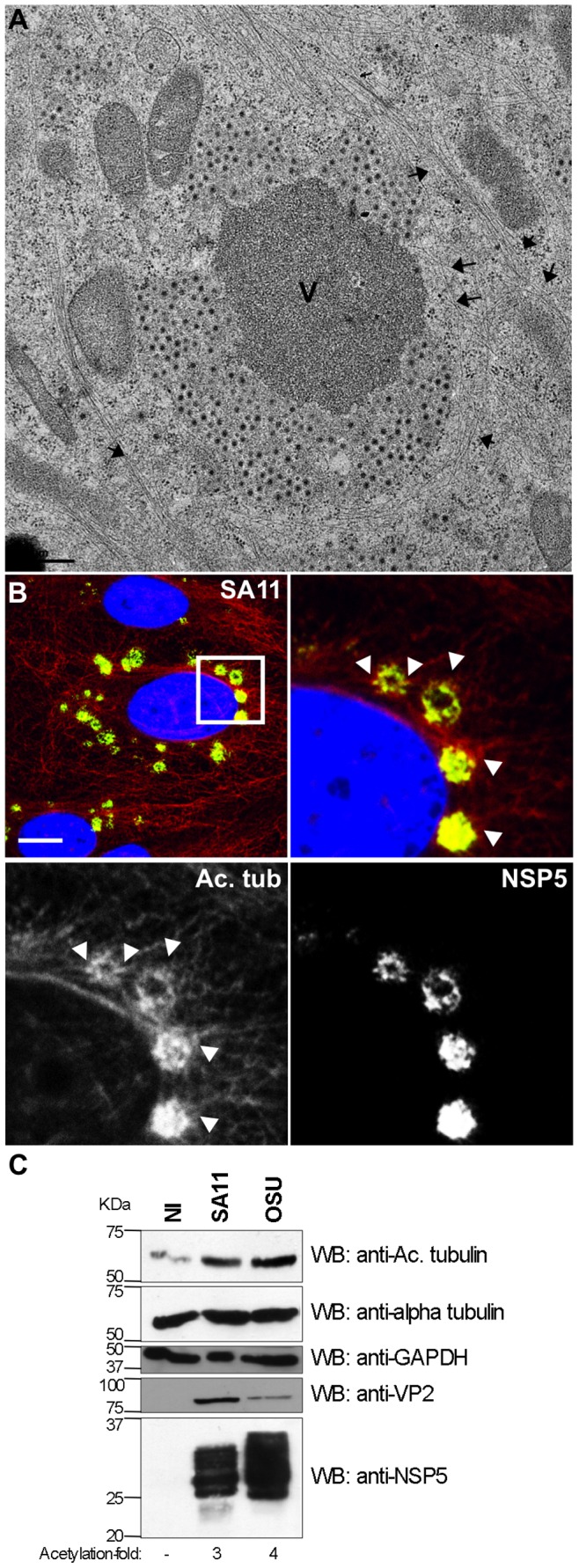
Viroplasms stabilize the MT-network. (**A**) Electron microscopy of SA11-infected MA104 cells at 8 hpi, showing viroplasm. Samples were frozen at high pressure, substituted and embedded in epon for MT detection. Black arrowheads indicate the MT-bundles; viroplasms (V). Scale bar is 0.5 µm. (**B**) Immunofluorescence of SA11-infected MA104 cells at 6 hpi showing viroplasms (anti-NSP5, green), acetylated tubulin (mAb anti-acetylated tubulin, red) and nucleus (DAPI, blue), upper left image. The white-boxed area shows an enlarged photomicrograph indicating the localization of the hyper-acetylated MTs (white arrowheads) in the viroplasm region. Scale bar is 15 µm (**C**) Immunoblotting of MA104 cell lysates from non-infected (NI) and SA11- or OSU-infected for 6 hpi [MOI; 25 VFU/cell]. Acetylated tubulin and total tubulin were detected with mAbs specific for acetylated alpha tubulin and alpha tubulin, respectively. Viral infection was detected with anti-NSP5 and anti-VP2 antibodies. GAPDH staining was used as loading control. The molecular weights (kDa) of the proteins are indicated.

In order to investigate whether viroplasms stabilize the MT-network, we performed indirect immunofluorescence and high-resolution electron microscopy using rotavirus proteins NSP5 and VP6 as viroplasm markers (Figure S6A). As shown in Figure 5A and Figures S6 B and C, it was possible to discern MT bundles adjacent to viroplams, suggesting a stabilization of the MT-network. To test this hypothesis, we analyzed the presence of acetylated-MT levels, which is a common marker for MT-stabilization [49]. By examination of the distribution of acetylated-MT by immunofluorescence, in mock infected (Figure S7A) and rotavirus SA11-infected cells (Figure 5B) we found higher levels of acetylated-MT levels in virus-infected cells. Moreover, viroplasms (detected with anti-NSP5 (green)) were colocalized by acetylated-MT (detected by a MAb anti-acetylated alpha tubulin (red)) (Figure 5B). Similar results were obtained in MA104 cells and CV-1 cells infected with simian rotavirus SA11 (Figure S7B). To quantify the difference in acetylated-tubulin levels in mock-infected or SA11- or OSU-infected cells, cell lysates were prepared at 6 hpi and the amount of acetylated-tubulin relative to total alpha-tubulin was assessed by immunoblotting. The lysates from SA11- and OSU-infected cells contained, respectively, three- to fourfold more acetylated-tubulin than the mock-infected cells. By contrast, the amounts of total alpha-tubulin were similar in all lysates (Figure 5C). These results suggest that rotavirus infection induces tubulin acetylation, thereby stabilizing the MT-network.

### Viroplasms are Embedded by Acetylated MTs

**Figure 6:**
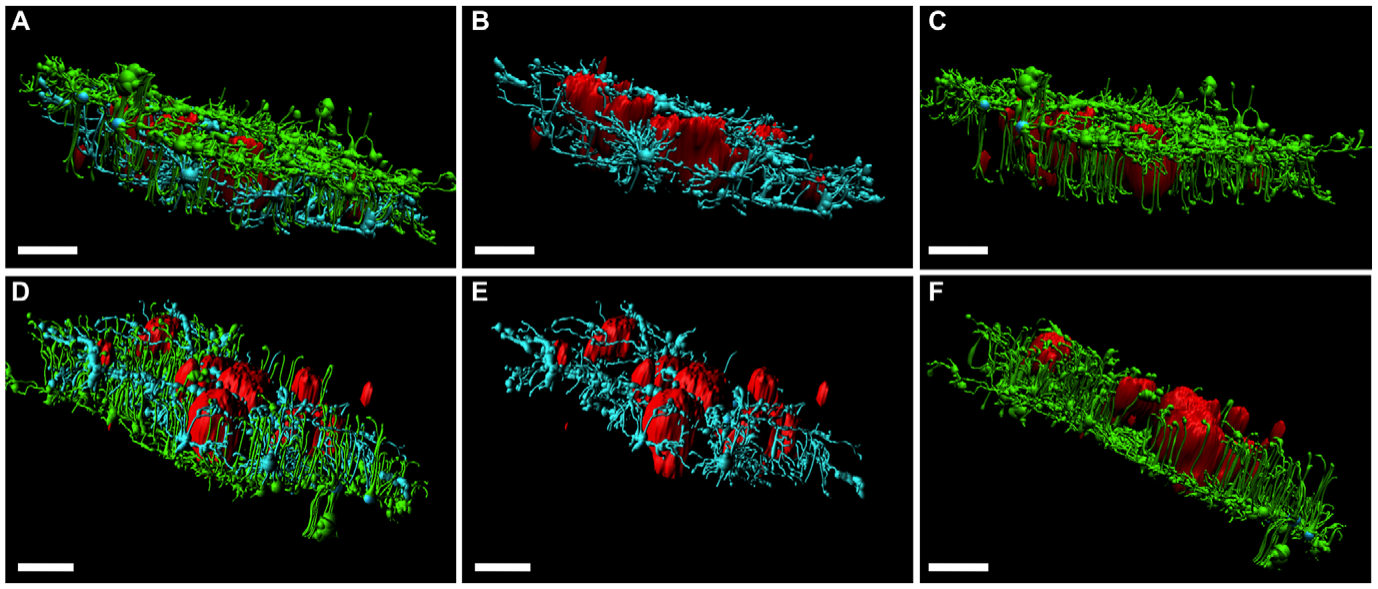
Viroplasms are embedded by acetylated-MTs. At 6 hpi, SA11-infected CV-1 cells [MOI; 25 VFU/ml] were fixed with methanol and immunostained for detection of viroplasms in red (anti-NSP5 antibody followed by a secondary antibody conjugated to Alexa 594), acetylated-MTs in cyan (mouse mAb anti-acetylated alpha-tubulin followed by a secondary antibody conjugated to Alexa 647), MTs in green (mouse mAb anti-alpha tubulin directly conjugated to Atto 488 (green)) and nuclei in blue (DAPI). Z-stack images were acquired by high-resolution CLSM and subsequently, 3D-reconstructions were performed using surface and filament algorithms from the surpass model of the Imaris 7.0 software (Bitplane, Switzerland). 3D-reconstructions are visualized from the topside (**A, B** and **C**) and the bottom side (**D, E** and **F**) of the preparation. Images show viroplasms (from **A** to **F**), acetylated-MTs (**A, B, D** and **E**) and MTs (**A, C, D** and **F**). Scale bars are 5 µm.

The stabilization of the microtubules by means of acetylation seems to play a role in the formation and maintenance of viroplasms. To thoroughly investigate the distribution of acetylated-MTs with respect to viroplasms, three-color immunofluorescence for viroplasms (Alexa 594, red), alpha tubulin (Atto 488, green) and acetylated-tubulin (Alexa 647, cyan) was carried out in SA11-infected MA104 cells at 6 hpi. After CLSM image acquisition, the obtained images were superimposed for a three-dimensional (3D) reconstruction of the viroplasms and the surrounded MTs with the surpass analysis imaging (Imaris software) (Video S3). In figure 6, viroplasms (red) from the top (Figures 6 A, B and C) and the bottom (Figures 6 D, E and F) sides of the preparation juxtaposed with MTs (green) and acetylated-MTs (cyan) are shown. Interestingly, viroplasms appear to be embedded by acetylated-MTs (Figures 6 C and F), while non-acetylated microtubules were found mostly in the cell topside of the preparation (Figures 6 A, B, D and E). These results support the hypothesis that the MT-network stabilized by tubulin acetylation is involved in the structure and dynamics of viroplasms.

### MTs and Kinesin Molecular Motor in Viroplasms Assembly

**Figure 7:**
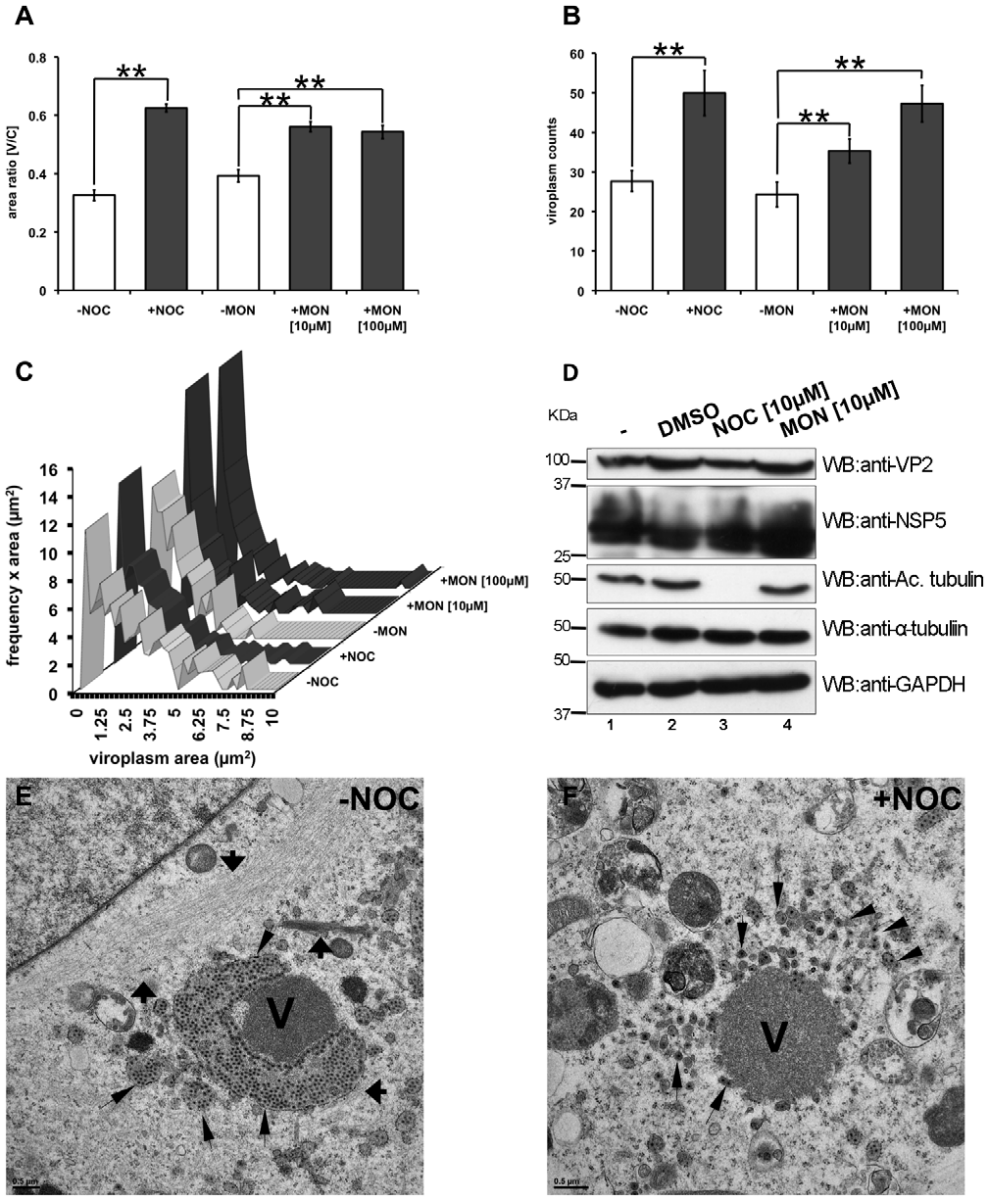
Perinuclear condensation and assembly of viroplasms involves MTs and kinesin molecular motor. At 1 hpi, SA11-infected NSP5-EGFP/MA104 cells [MOI; 25 VFU/cell] were treated with nocodazole [10 µM] or monastrol [10 or 100 µM] or untreated (2% DMS0). Cells were fixed at 6 hpi and stained for plasma membrane and nucleus. The perinuclear condensation [V/C ratio] (**A**) and the number per cell (**B**) of viroplasms were plotted for each drug treatment. Data is presented as mean±SEM; t-test, (**) p<0.01; number >60 cells (**C**) Distribution of the viroplasms frequency upon drugs treatment. d) Immunoblotting of lysates of untreated cells (lanes 1 and 2) or cells treated with either nocodazole (lane 3) or monastrol (lane 4) treatment. Viral infection was detected with an anti-VP2 and anti-NSP5 specific antibodies; acetylated-tubulin and tubulin were detected with a mAb anti-acetylated alpha tubulin and a mAb anti-alpha tubulin, respectively; detection of GAPDH was used as loading control. Protein molecular weights are indicated. Electron microscopy of viroplasms from SA11-infected MA104 cells [MOI of 250 VFU/cell] fixed with 2.5% GA at 6 hpi; cells were untreated (**E**) or treated with 10 µM nocodazole (**F**) for 1 hpi. Viroplasms (V) is indicated, and thick black arrows correspond to MT-bundles and thin black arrows correspond to ER. Scale bars, 0.5 µm.

We reasoned that if MTs contribute to the structure and dynamics of viroplasms, then the absence of polymerized MTs as well as the inhibition of MT molecular motors should prevent viroplasm fusion and their perinuclear re-localization. To test this hypothesis, SA11-infected MA104 cells were treated at 1 hpi with nocodazole to depolimerize-MTs or monastrol, an allosteric inhibitor of the Eg5 kinesin family [50]. Cells were then fixed at 6 hpi and stained to determine perinuclear localization (ratio [V/C]) and to count viroplasms. As expected, in presence of nocodazole viroplasms were unable to move to the perinuclear region and to fuse, as reflected by a consistently high number of viroplasms per cell. Interestingly, upon treatment with 10 or 100 µM monastrol viroplams were unable to move to the perinuclear region and, in a dose-dependent manner, to reduce the viroplasms number (Figures 7 A and B). Additionally, when frequencies of viroplasms size were plotted upon nocodazole or monastrol treatment, formation of large structures (>2 µm^2^) was not observed, with a prevalence of small size viroplasms (0.025 to 2 µm^2^) (Figure 7C). Upon drug treatments, identical relative amounts of host markers, such as tubulin and GAPDH, and viral infection markers, such as NSP5 and VP2 were detected by immunoblotting. As expected, with nocodazole treatment no acetylated tubulin was observed (Figure 7D). To confirm this data, electron microscopy of viroplasms from virus-infected cells treated with nocodazole under the same conditions as described above was performed. In the untreated control cells, it was possible to distinguish viroplasm as well as the MT-bundles (Figure 7E). In contrast, while the internal domain seemed to remain intact upon nocodazole treatment, the ER surrounding the viroplasms appeared completely disrupted. As expected, in nocodazole treated cells MTs remained invisible (Figure 7F). Similar data was obtained by co-immunostaining in which, upon nocodazole treatment, VP2 and VP6 appeared disperse around the viroplasm internal domains of the viroplasms and in the cell cytosol, which was in marked contrast to the punctuated localization adjacent to the viroplasm (detected via anti-NSP5) observed in control cells (Figure S8). These results strongly suggested that MTs and kinesin from the Eg5 family were necessary for the assembly of viroplasms as well as for their re-localization in the perinuclear region of the cells.

### Viroplasm Structure and Perinuclear Condensation are Maintained in a MT and Kinesin Eg5-dependent Manner

**Figure 8:**
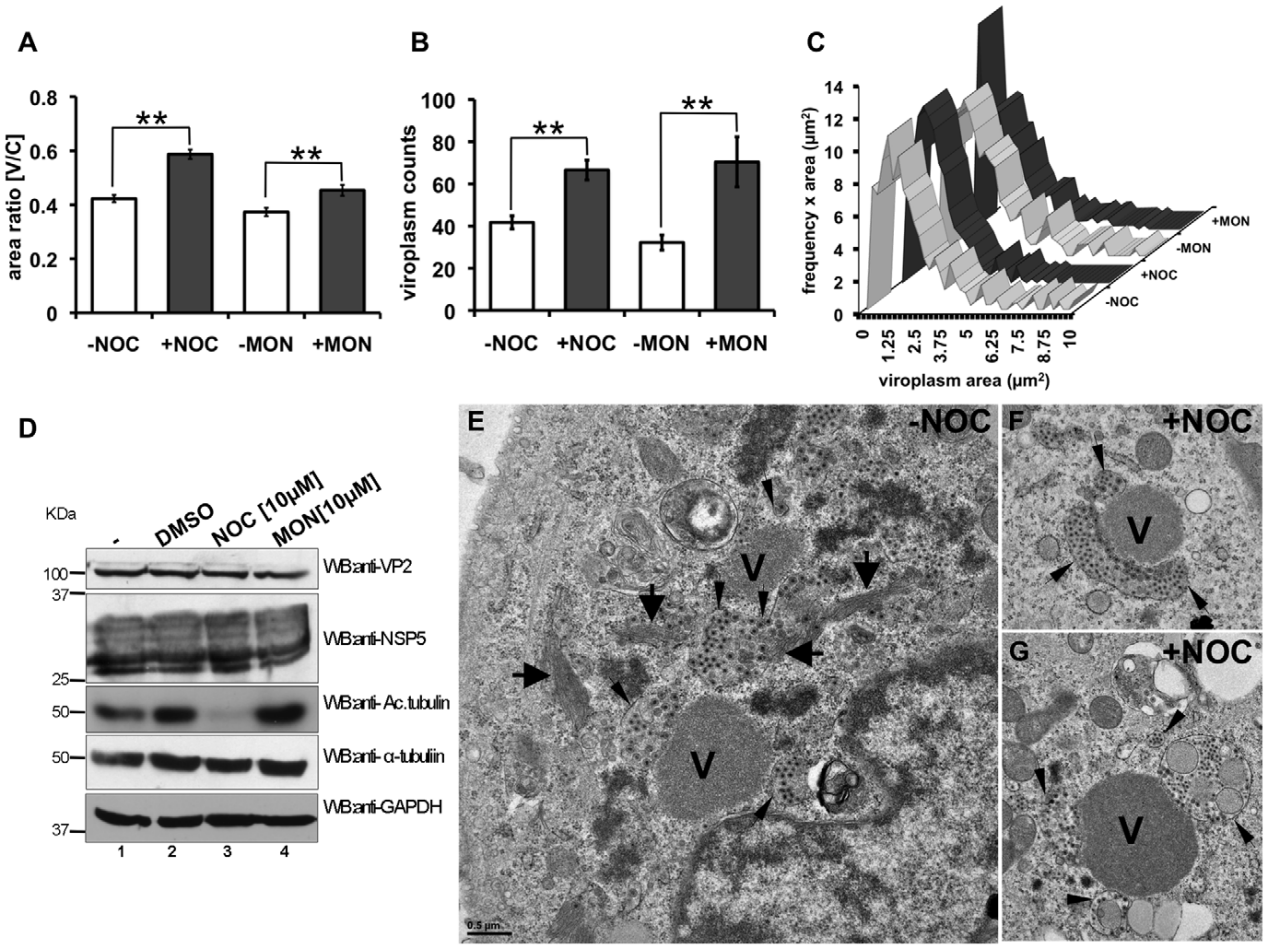
Structure and perinuclear condensation of viroplasms are maintained in an MT/kinesin-dependent manner. At 5 hpi, SA11-infected NSP5-EGFP/MA104 cells [MOI; 25 VFU/cell] were treated with 10 µM of either nocodazole or monastrol. Cells were fixed at 6 hpi and stained for plasma membrane and nucleus. The perinuclear condensation [V/C ratio] (**A**) and the numbers per cell (**B**) of viroplasms were plotted for each drug treatment. Data is presented as mean±SEM; t-test, (**) p<0.01; number >60 cells. (**C**) Distribution of the viroplasms frequency upon drugs treatment. (**D**) Immunoblotting of SA11-infected cell lysates in absence of drug treatment (lanes 1 and 2) or treatment with either nocodazole (lane 3) or monastrol (lane 4) treated. Viral infection was detected with an anti-VP2 and anti-NSP5 specific antibodies; acetylated-tubulin and total tubulin were detected with mAbs anti-acetylated alpha tubulin and anti-alpha tubulin, respectively; staining of GAPDH was used as loading control. Protein molecular weights are indicated. Electron microscopy of viroplasms from SA11-infected MA104 cells [MOI; 250 VFU/cell] fixed with 2.5% GA at 6 hpi. At 5 hpi, cells were untreated (**E**) or treated with nocodazole [10 µM] (**F** and **G**). Viroplasms (V) were labeled, thick black arrows indicate MT-bundles and thin black arrows indicate ER. Scale bars are 0.5 µm.

In the present study, we have presented evidence that the viroplasm dynamics (fusion and perinuclear condensation) required both the stabilization of MTs as well as the Eg5-kinesin motor. We next asked whether the MT-network and the kinesin-molecular motors were necessary for the structural maintenance and localization of viroplasm once they had formed and localized to the perinuclear region. To address this question, we performed experiments in nocodazole or monastrol treated and virus-infected cells at 5 hpi, a time point in which viroplasms were already formed and perinuclearly localized. After 1 h of treatment, both drugs, mediated the decondensation of viroplasms from the perinuclear area and an increment in viroplasms number (Figures 8 A and B). Moreover, viroplasms showed a tendency to diminish in size as denoted by the analysis of size frequencies upon drug treatment (Figure 8C). Similar relative amounts of host markers including tubulin and GAPDH, and viral proteins such as NSP5 and VP2 were detected by immunoblotting. As expected, no acetylated tubulin was detected in cellular lysates treated with nocodazole (Figure 8D). Electron microscopy inspection performed under the same conditions, revealed a detachment of viroplasm from the ER. Interestingly, the presence of vacuole-like structures was observed when compared with the untreated controls (Figures 8 E, F and G). These data indicated that the MT-network and the kinesin Eg5 family are playing active roles in controlling both, the spatial positioning of viroplasms within the cell and the maintenance of their structure.

### NSP2 Mediates VLS Fusions While VP2 Mediates VLS Perinuclear Localization

**Figure 9:**
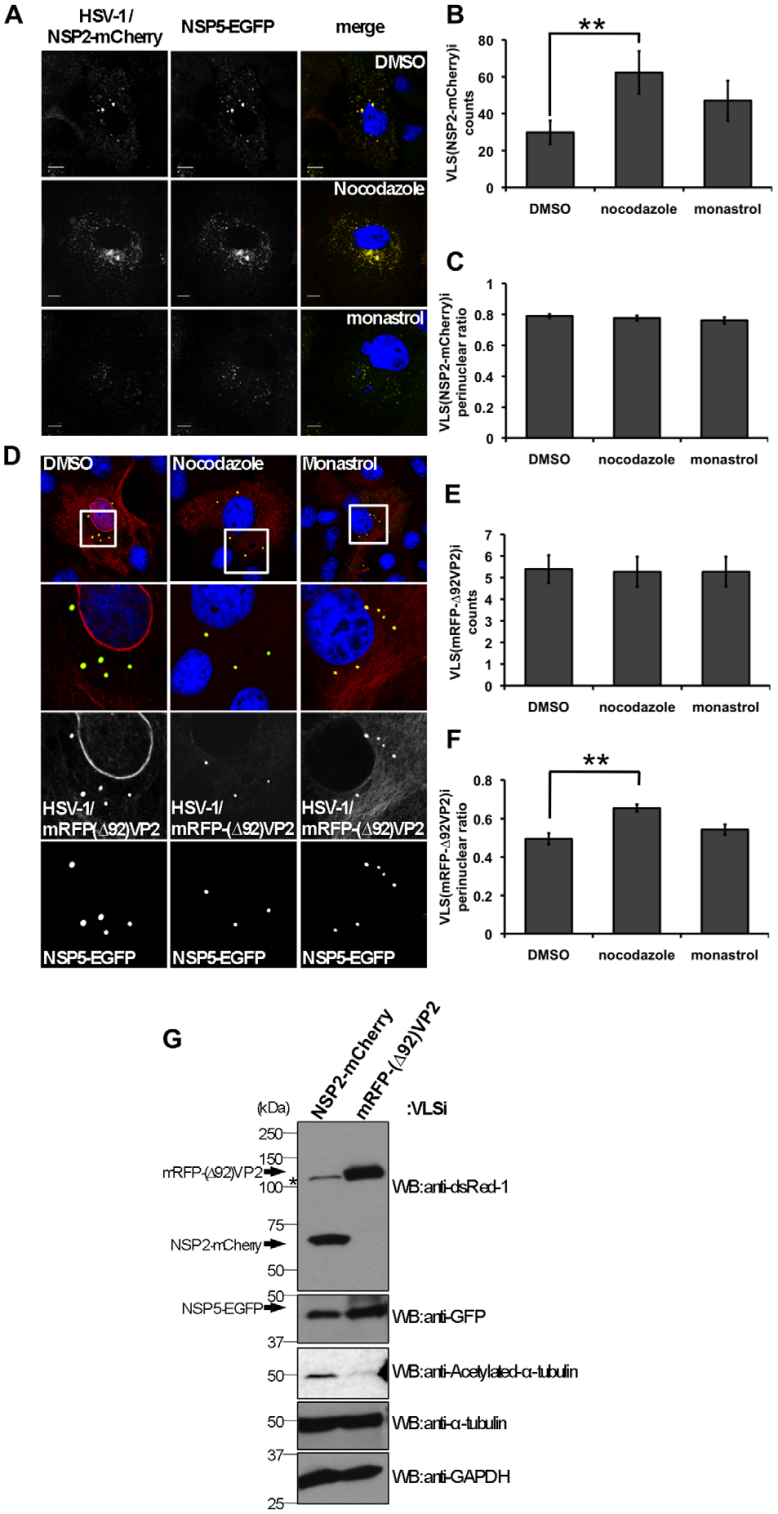
VLS induced by NSP2 and VP2 are MT-dependent and have different roles. HSV-1 amplicon vectors [MOI; 1TU/cell] for NSP2-mCherry (**A**) or mRFP-Δ92VP2 (**D**) were used to infect NSP5-EGFP/MA104 cells. Cells were treated for 1 h with 10 µM of either nocodazole or monastrol before fixation (at 24 hpi) and nuclei stained with DAPI (blue). Scale bar is 10 µm. The numbers of VLS-NSP2i and VLS-VP2i per cell (**B** and **E**) and their perinuclear localization (**C** and **F**) was plotted for each drug treatment. Data is presented as mean±SEM; t-test (**) p<0.01, n>40 cells. (**G**) Immunoblotting from cell lysates of NSP5-EGFP/MA104 cells infected with HSV-1 amplicons for NPS2-mCherry or Δ92VP2-mRFP [MOI, 1TU/cell] for 24 h. The viral fusion proteins were detected with a mAb anti-dsRed1 (NSP2 and VP2) and NSP5-EGFP was detected with a mAb anti-GFP. *, correspond to an inespecific band also present in mock transfection. Acetylated tubulin and total tubulin were determined with mAbs anti-acetylated alpha-tubulin and anti-alpha-tubulin, respectively. Staining of GAPDH was used as loading control. The protein molecular weights (kDa) are indicated.

To gain further insight into the role of viral proteins in viroplasms fusion and viroplasm perinuclear localization, we engineered an HSV-1 amplicon [45] encoding NSP2 or Δ92-VP2 [40] and used them to transduce NSP5-EGFP/MA104 cells in order to induce the formation of a consistent number of viroplasm-like structures (VLS), VLS(NSP2)i or VLS(Δ92-VP2)i, respectively. Using this experimental setting, we monitored the behavior of VLS(NSP2)i or VLS(Δ92-VP2)i upon treatment with nocodazole or monastrol for 1 h before fixation. Both, VLS(NSP2)i and VLS(Δ92-VP2)i, appeared to be sensitive to nocodazole treatment but in a different manner. When compared to mock treated samples the number of VLS(NSP2)i increase upon nocodazole treatment, while the number of VLS(Δ92VP2)i did not (Figures 9 B and E). Conversely, VLS(Δ92VP2)i, but not VLS(NSP2)i, was able to delocalize from the perinuclear area upon nocodazole treatment (Figures 9 C and F). None of the VLSs were sensitive to treatment with monastrol. Additionally, acetylated-tubulin levels were higher in cellular extracts obtained from VLS(NSP2)i than from VLS(Δ92VP2)i (Figure 9G). These data suggest complementary roles for NSP2 and VP2 in the dynamics of viroplasms.

## Discussion

As part of their infective strategy, some viruses have the ability to subvert the MT transport system of the cell in order to facilitate their replication and to enhance their spread into surrounding cells and tissues [51]. There are numerous examples in the current literature linking the MT-network with trafficking of viral particles, considered as cargoes, which move to opposite ends of MTs to their replication sites immediately after cell entry or to move the newly assembled viral progeny to the plasma membrane. The two most common molecular motors involved in viral trafficking are dyneins and kinesins [52], [53], [54]. Many viruses utilize cytoplasmic dynein to facilitate their movement towards the microtubule-organizing centre (MTOC) during initial establishment of the infection as is the case for adenoviruses [55], [56], adeno-associated viruses [57], african swine fever virus [58], canine parvovirus [59], herpes viruses (HSV-1, PRV) [60], [61], [62], rhaboviruses (lyssavirus, rabies virus) [63], [64] and retroviruses (HIV-1, foamy virus) [65], [66]. Multiple members of the kinesin superfamily are also involved in viral trafficking; being the best characterized ones are those corresponding to kinesin-1, which have been directly related to the anterograde transport concerning vaccinia viruses [67], [68], [69] and herpes viruses [70], [71], [72], [73], [74]. However, little is known about the role of the host cytoskeleton in the formation and dynamics of cytosolic viral factories, like the ones formed by reoviridae members, such as orbiviruses, reoviruses and rotaviruses [29], [30], [31], [35]. In the present work, we present for the first time direct evidence that rotavirus viroplasms are dynamic structures during the virus replicative cycle. We demonstrate using time-lapse confocal microscopy, high-resolution electron microscopy and viroplasm quantification for size and number that viroplasms are able to perform at least two different processes: viroplasm-viroplasm fusion and movement towards the perinuclear region of the cell. These dynamic processes involve the microtubular network at multiple steps in which MTs get stabilized in association with tubulin acetylation and formation of MT-bundles around viroplasms. Interestingly, using 3D modelling from confocal microscopy, we determined that viroplasms are embedded by acetylated-MTs. These results are shared by different viral strains and cell lines tested, strongly suggesting a generalized characteristic of rotavirus viroplasms. Moreover, rotavirus viroplasms are not unique among the reoviridae members in subverting the MT-network by stabilization. The reoviral protein µ2 (like the one from strain T1L), a minor core protein and a component of reovirus viral factories, is able to bind MTs directly and to stabilize them through acetylation. This association is fundamental to promote the fibrillar morphology to the reoviral factories [35].

We observed a rather constant formation of small viroplasms during viral infection, which could be the result of continuous viral protein synthesis [30]. Nevertheless, our findings demonstrate that the enlargement of the individual viroplasms does not dependent solely on the incorporation of newly synthesized viroplasm proteins but also on the fusion of these structures. Our data supports the conclusion, that viroplasm-fusion contributes significantly to their enlargement and reduction in number. Interestingly, recent studies have suggested that lipid droplets [75] as well as proteins related to unfolded protein response [76] localize in viroplasms, proposing the viroplasm as a regulator of cellular components by a process involving host subvertion. These observations are in agreement with our data, since we cannot discard the contribution of other host components for the stabilization and dynamics of the viroplasms.

Viroplasms are composed of internal and external domains [21], [22], [23], [77]. Upon nocodazole treatment the ER was dissociated from the viroplasms, probably because of alterations in the external VP6-rich domain, suggesting that the MTs have a role in maintaining the correct interactions of viroplasms with other components of the cell. We can speculate that viroplasm components present in the external domain can associate directly or indirectly with the components of the MT- network.

We also present evidence suggesting that viroplasm assembly, structural maintenance and juxtanuclear-localization depends not only on an intact and stabilized MT-network as well as on Eg5-kinesin. This is a surprising result since, Eg5 (also called kinesin-5) is commonly associated with spindle pole separation and spindle bipolarity at the initiation of mitosis. Eg5 is usually found in an inactive form in the cytosolic compartment during the interphase [52]. In unifected cells, direct activation of Eg5 by the phosphorylation of the tail domain by cyclin-dependent kinase-1, increases its ability to bind MTs and the spindle body. Upon rotavirus infection cells become arrested in S phase (unpublished data) and do not undergo apoptosis at early time post-infection [78], [79], [80], [81], thus suggesting that rotavirus required an integral MT-network during the replicative cycle. One possibility is that rotavirus infection activates Eg5 through a process that most likely involves phosphorylation [82], [83], although this hypothesis remains to be tested. We cannot rule out at present, that other molecular motors, such as other kinesins or dyneins also participate in viroplasm dynamics. Through a simplified model of viroplasm assembly (formation of VLS induced by NSP2 or VP2 in the presence of NSP5), we established that both viral proteins participate in the viroplasm dynamics but in a different manner. Accordingly, this data is consistent with previous observations performed by the group of Poncet [31] in which NSP2 was shown to associate with free and polymerized tubulin. Even more interesting it is the recent finding that the C-terminal helix of NSP2 is an open conformation with a swapping domain for protein interaction that may be important in viroplasm formation [84]. Altogether, these data are consistent in implicating NSP2 in a direct role in viroplasm-fusion. We used the N-terminal deletion of VP2 version, Δ92-VP2, which was shown to assemble with itself and to associate with other viral proteins in non-infected cells [85], [86]. HSV-1 amplicon coding for Δ92-VP2 has been previously used [40] and our own unpublished data indicate that the full-length or the deleted version are equally able to produce VLS when transduced in NSP5-EGFP/MA104 cell line. Through a mechanism that remains yet to be elucidated we show here that VP2 plays a role in the localization of the VLS to the perinuclear area. The VLS (Δ92-VP2)i translocation could depend on a direct interaction of VP2 with a kinesin-like molecular motor. Alternatively, it may depend on an indirect interaction through a complex involving NSP5, an essentially disordered protein that could be structurally modified by VP2 to adopt a favorable spatial conformation. The fact that Δ92-VP2 allowed the movement of the VLS to the perinuclear area highly suggests that this portion of the N-terminal is not involved in VLS formation or in perinuclear condensation. Moreover, neither VLS (NSP2)i nor VLS (Δ92-VP2)i were sensitive to monastrol treatment indicating that another viroplasm component must be in association with the Eg5-kinesin.

In summary, we showed that rotavirus viroplasms can fuse and condensate to the perinuclear area of the cell and that the MT-network has an essential role in these processes. In addition, virus infection induces MT-network stabilization by inducing tubulin acetylation. The definitive links between these observations to the viral replicative cycle are still open and are the subject of current investigations in our laboratory.

## Supporting Information

Figure S1 **Viroplasm measurements of SA11-infected NSP5-EGFP/MA104 cells.** Plot of the number (and mean area (µm^2^) (O) of viroplasms per cell, determined at various times post-infection. Data is presented as mean±SEM, n>60 cells.
(TIF)

Figure S2 **Time-lapse confocal microscopy of rotavirus SA11-infected NSP2-EGFP/MA104 cells [MOI, 25 VFU/cell].** Acquisition was performed in an infection period from 5 to 24 hpi. (**A**) The most representative frames are shown. Clusters of viroplasms before fusion are indicated in numerated white boxes; a red arrow points the fused viroplasms. (**B**) Enlarged images of fusion occurring in white boxes #2 (upper panel) and # 6 (lower panel) are shown. A red arrow indicates the fused viroplasms.
(TIF)

Figure S3 **Rotaviruses rearrange the host cytoskeketon**. CV-1 (upper panel) and MA104 (lower panel) cells were infected with simian rotavirus SA11 or porcine rotavirus OSU [25 VFU/cell]. At 6 hpi, cells were fixed with methanol and immuno stained for actin (rabbit polyclonal anti-actin, left colums); MT (mouse mAb anti-alpha tubulin, middle columns) and intermediate filaments (rabbit polyclonal anti-vimentin). Merge with viroplasms is shown for each cytoskeleton-network component. Viroplasms were immunostained with a guinea pig polyclonal anti-NSP5 antibody, red or green and actin and vimentin were detected in red and MTs were detected in green. Non-infected cells (mock) are shown. The green arrow indicates the MT-bundles surrounding viroplasms. Scale bars are 20 µm.
(TIF)

Figure S4 (**A**) SA11-infected CV-1 cells [MOI; 10 mVFU/cell], were treated at 1hpi with 10 µM of nocodazole, vinblastine or taxol. At 6 hpi, the cells were fixed and stained for viroplasms (anti-NSP5 followed by a secondary conjugated to Alexa 488, green) plasma membrane (WGA-Alexa 594, red) and nuclei (Hoechst 33342, blue). The total area of the cell (yellow line), the area of the cell where viroplasms are distributed (green line) and nucleus (blue line) are shown. Scale bar is 10 µm. OSU-infected CV-1 cells [MOI; 25 VFU/cell] treated at 1 hpi with 10 µM nocodazole, vinblastine or taxol. At 6 hpi, cells were fixed and stained for viroplasms, plasma membrane and nuclei. The perinuclear condensation of viroplasms (**B**) and the numbers of viroplasms per cells (**C**) were determined upon each drug treatment. Data is presented as mean ± SEM; t-test, (**) p<0.01, N≥60.
(TIF)

Figure S5 **Rotavirus replication is not affected by nocodazole treatment. (A**) Viral fitness curve of simian rotavirus SA11-infection in treated and non-treated CV-1 cells [MOI; 25 VFU/cell]. At 1 hpi, cells were untreated ( ) (2% DMSO) or treated with 10 µM nocodazole ( and cells were harvested at 2, 4, 6, 8, 12 and 24 hpi. The viral titers (VFU/ml) for each time point were determined using NSP5-EGFP/MA104 and were plotted. (**B**) SA11-infected CV-1 cells [MOI; 25 VFU/cell] were treated at 1 hpi, with 10 µM nocodazole. At 18 hpi, cells were harvested, virus genome was extracted, resolved in a 10% SDS-PAGE and silver stained to detect dsRNA genome segments.
(TIF)

Figure S6 **Immunofluorescence analysis of viroplasms in SA11-infected CV-1 cells [MOI; 25 VFU/cell] at 6 hpi.** (**A**) The viroplams internal and external (white arrows) domains were detected with specific anti-NSP5 serum (green) and with mAb anti-VP6 antibody (red), respectively. (**B**) Co-immunofluorescence analysis of SA11 viroplasms stained with a specific anti-NSP5 serum (red) and MTs stained with mAb anti-tubulin conjugated to Atto 488 (green). (**C**) Co-immunofluorescence analysis of SA11 viroplasms stained with mAb anti-VP6 (red) and MTs stained with mAb anti-tubulin conjugated to Atto 488 (green). The white-boxed area shows the site for picture enlargement in which MT-network (white arrows) co-localize with viroplasms. Scale bars are 10 µm.
(TIF)

Figure S7 **Acetylated MTs in SA11-infected CV-1 cells.** (**A**) Immunostaining for acetylated MTs (mAb anti- acetylated alpha-tubulin, red) of MA104 (left) and CV-1 (right) cells. (**B**) Immunofluorescence analysis of SA11-infected CV-1 cells at 6 hpi, showing viroplasms (anti-NSP5, green), acetylated tubulin (mAb anti-acetylated tubulin, red) and nuclei (DAPI, blue), upper image left. The white-boxed area shows an enlarged image that indicates the localization of the hyper-acetylated MTs (white arrowheads) in the viroplasm region. Scale bars are 10 µm.
(TIF)

Figure S8 **Viroplasms show a disperse morphology upon nocodazole treatment.** Rotavirus SA11-infected CV-1 cells [MOI; 25 VFU/cell] were untreated (left panel) or treated with 10 µM nocodazole (right panel) for 5 hours before fixation. At 6hpi, cells were fixed with methanol and co-immunostained for: (**A**) MT (mAb anti-alpha tubulin conjugated to atto 488, green) and VP2 (anti-VP2, red); (**B**) MT (mAb anti-alpha tubulin conjugated to atto 488, green) and VP6 (mAb anti-VP6, red); (**C**) VP2 (anti-VP2, red) and VP6 (mAb anti-VP6, green); (**D**) VP2 (anti-VP2, green) and NSP5 (anti-NSP5, red) and (**E**) NSP5 (anti-NSP5, green) and VP6 (mAb anti-VP6, red). In each panel, the left and middle columns show the separate acquisitions and the right column shows the merged images. Nuclei are stained with DAPI (blue). Scale bars are 10 µm. (TIF)
(TIF)

Table S1 **Primers for plasmid pHSV-1-NSP2-mCherry construction.**
(DOCX)

Video S1 **Confocal time-lapse microscopy of NSP5-EGFP/MA104 cells infected with simian rotavirus SA11 [MOI, 25 VFU/cell].** Cells were recorded from 4 to 20 hpi at a rate of one frame per 20 min. Yellow arrows indicate the site of viroplasm fusion. Scale bar is 10 µm.
(MP4)

Video S2 **Confocal time-lapse microscopy of NSP2-EGFP/MA104 cells infected with simian rotavirus SA11 [MOI, 25 VFU/cell].** Cells were recorded from 5 to 24 hpi at a rate of one frame per 20 min. Yellow arrows indicate the site of viroplasm fusion. Scale bar is 10 µm.
(MP4)

Video S3 **Animation of the 3D reconstruction from Z-stack showing viroplasms (red), MTs (green) and acetylated-MTs (cyan).** Scale bar is 5 µm.
(MP4)
