# Supplementary figures and images for: Rotavirus Viroplasm Fusion and Perinuclear Localization Are Dynamic Processes Requiring Stabilized Microtubules
Source: PLoS One. 2012 Oct 23;7(10):e47947. doi: 10.1371/journal.pone.0047947 (PMC3479128; doi:10.1371/journal.pone.0047947)

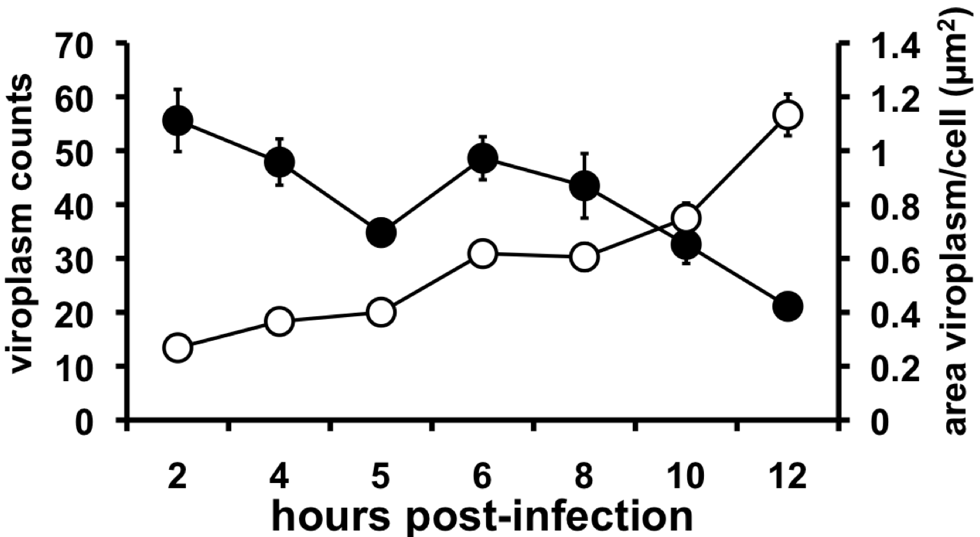

Supplement: Figure S1 — Viroplasm measurements of SA11-infected NSP5-EGFP/MA104 cells. Plot of the number (and mean area (µm2) (O) of viroplasms per cell, determined at various times post-infection. Data is presented as mean±SEM, n>60 cells. (TIF) [file pone.0047947.s001.tif]

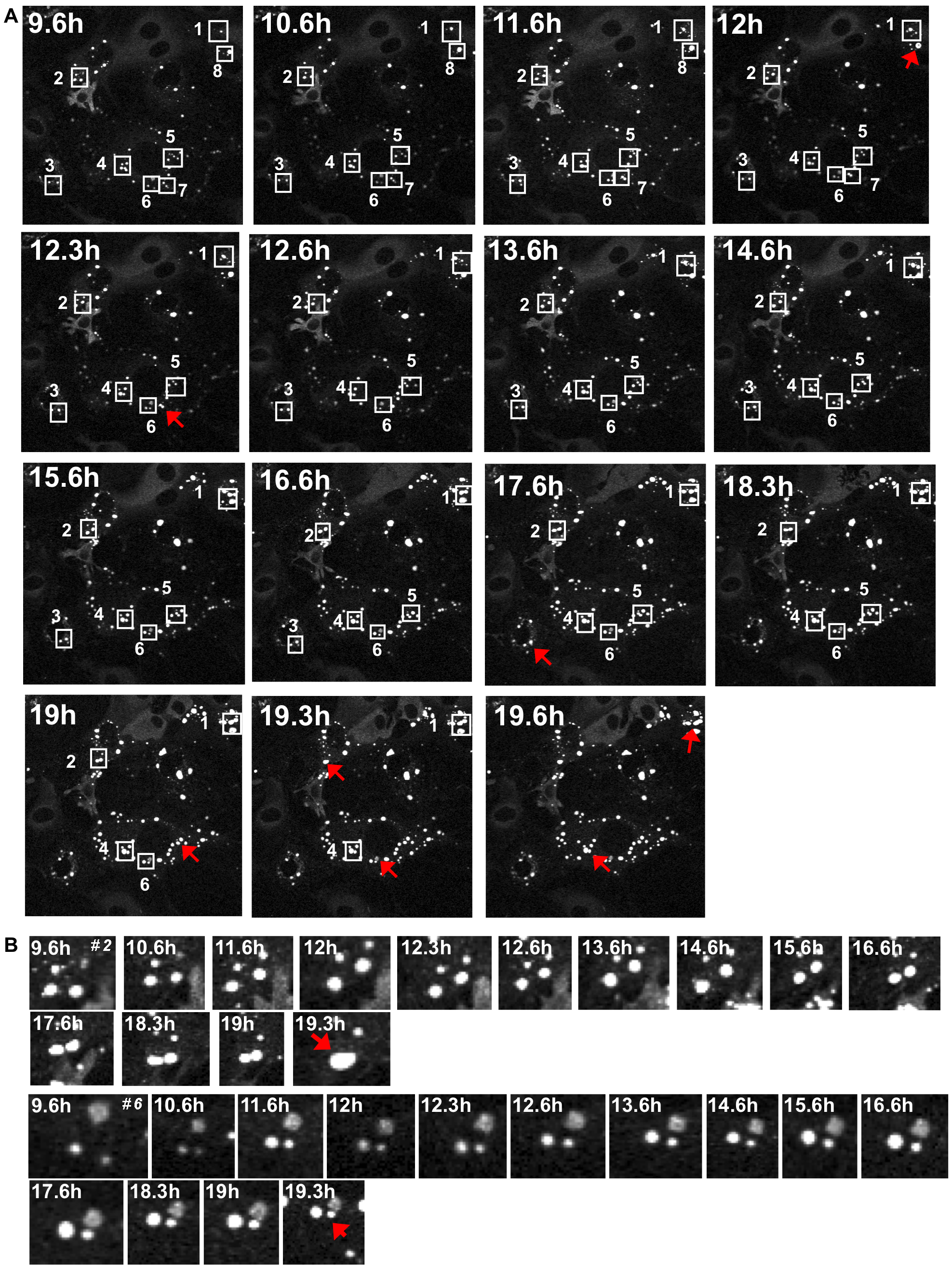

Supplement: Figure S2 — Time-lapse confocal microscopy of rotavirus SA11-infected NSP2-EGFP/MA104 cells [MOI, 25 VFU/cell]. Acquisition was performed in an infection period from 5 to 24 hpi. (A) The most representative frames are shown. Clusters of viroplasms before fusion are indicated in numerated white boxes; a red arrow points the fused viroplasms. (B) Enlarged images of fusion occurring in white boxes #2 (upper panel) and # 6 (lower panel) are shown. A red arrow indicates the fused viroplasms. (TIF) [file pone.0047947.s002.tif]

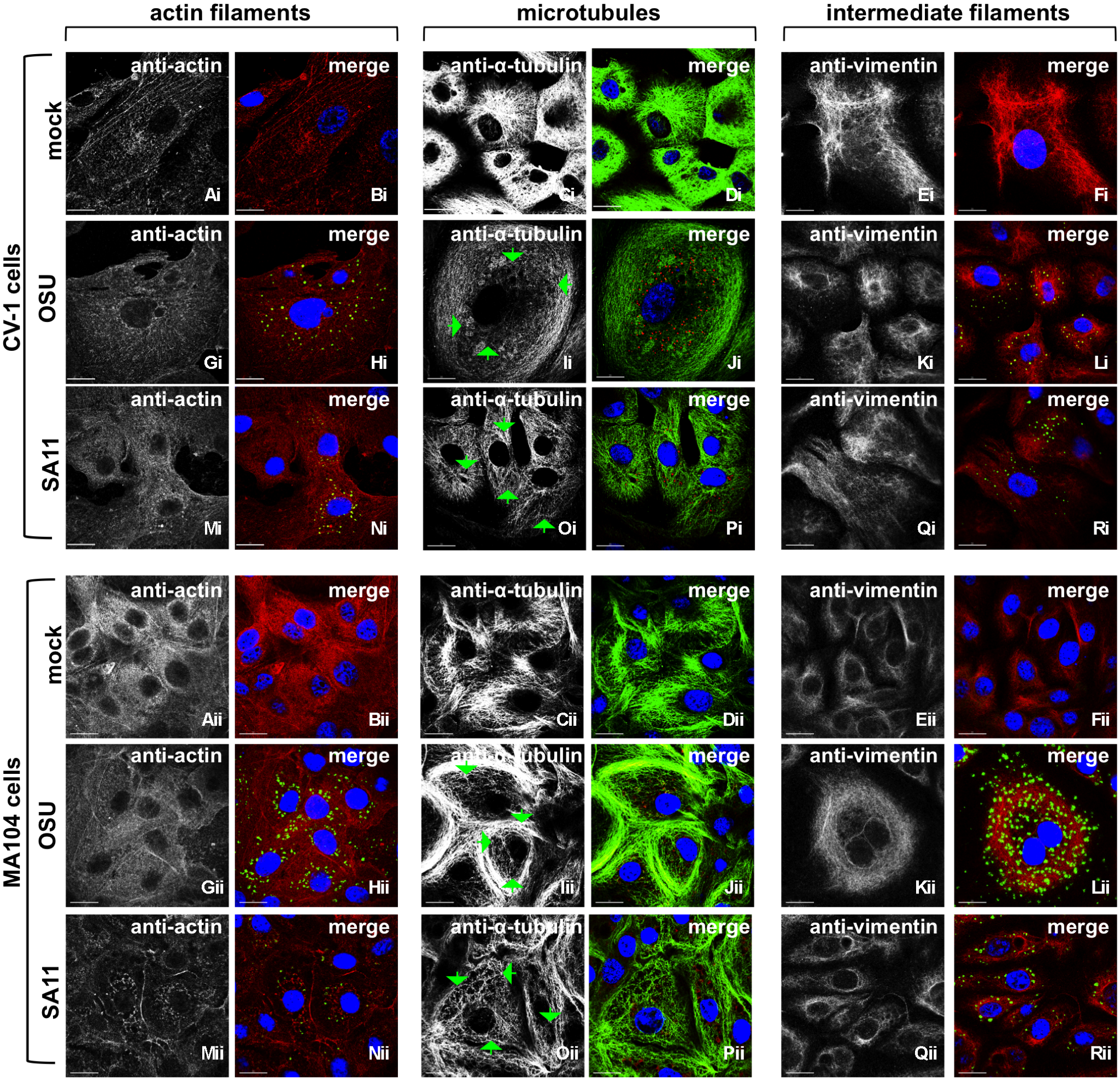

Supplement: Figure S3 — Rotaviruses rearrange the host cytoskeketon. CV-1 (upper panel) and MA104 (lower panel) cells were infected with simian rotavirus SA11 or porcine rotavirus OSU [25 VFU/cell]. At 6 hpi, cells were fixed with methanol and immuno stained for actin (rabbit polyclonal anti-actin, left colums); MT (mouse mAb anti-alpha tubulin, middle columns) and intermediate filaments (rabbit polyclonal anti-vimentin). Merge with viroplasms is shown for each cytoskeleton-network component. Viroplasms were immunostained with a guinea pig polyclonal anti-NSP5 antibody, red or green and actin and vimentin were detected in red and MTs were detected in green. Non-infected cells (mock) are shown. The green arrow indicates the MT-bundles surrounding viroplasms. Scale bars are 20 µm. (TIF) [file pone.0047947.s003.tif]

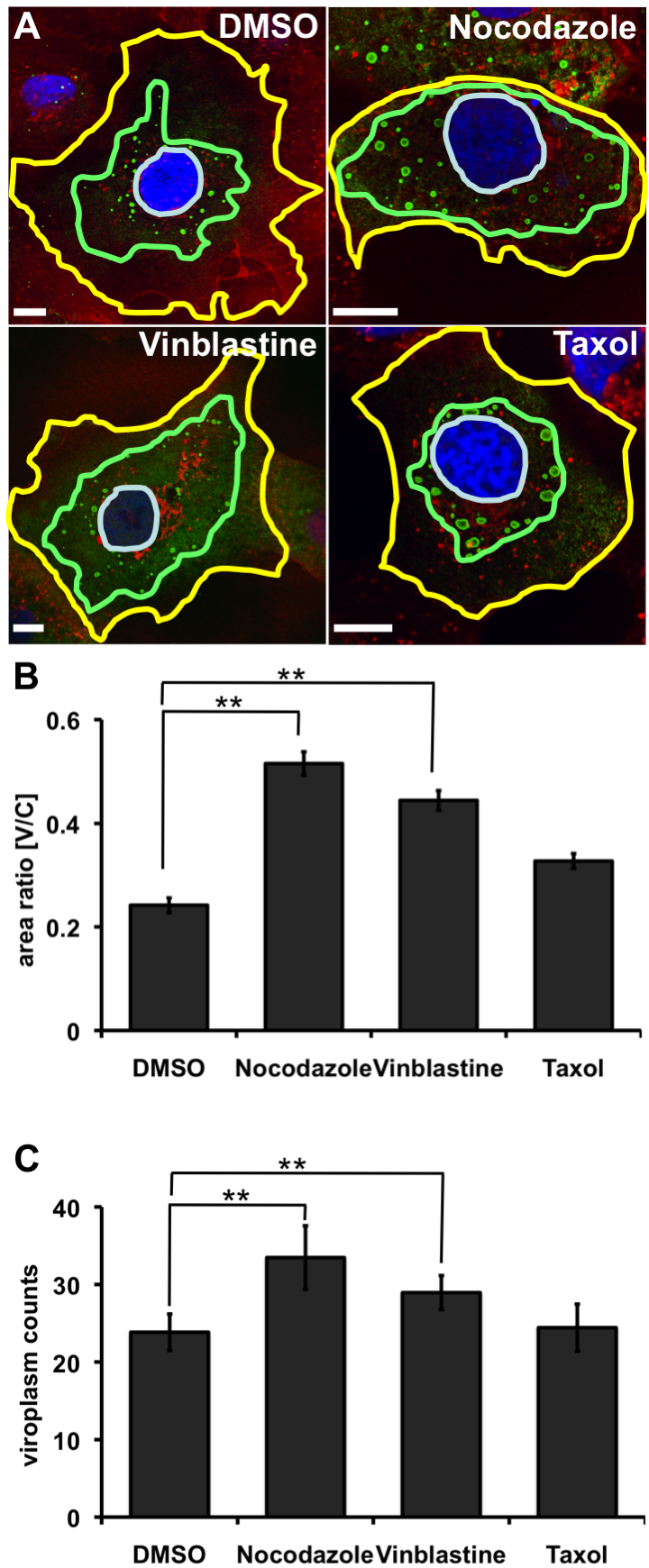

Supplement: Figure S4 — (A) SA11-infected CV-1 cells [MOI; 10 mVFU/cell], were treated at 1hpi with 10 µM of nocodazole, vinblastine or taxol. At 6 hpi, the cells were fixed and stained for viroplasms (anti-NSP5 followed by a secondary conjugated to Alexa 488, green) plasma membrane (WGA-Alexa 594, red) and nuclei (Hoechst 33342, blue). The total area of the cell (yellow line), the area of the cell where viroplasms are distributed (green line) and nucleus (blue line) are shown. Scale bar is 10 µm. OSU-infected CV-1 cells [MOI; 25 VFU/cell] treated at 1 hpi with 10 µM nocodazole, vinblastine or taxol. At 6 hpi, cells were fixed and stained for viroplasms, plasma membrane and nuclei. The perinuclear condensation of viroplasms (B) and the numbers of viroplasms per cells (C) were determined upon each drug treatment. Data is presented as mean ± SEM; t-test, (**) p<0.01, N≥60. (TIF) [file pone.0047947.s004.tif]

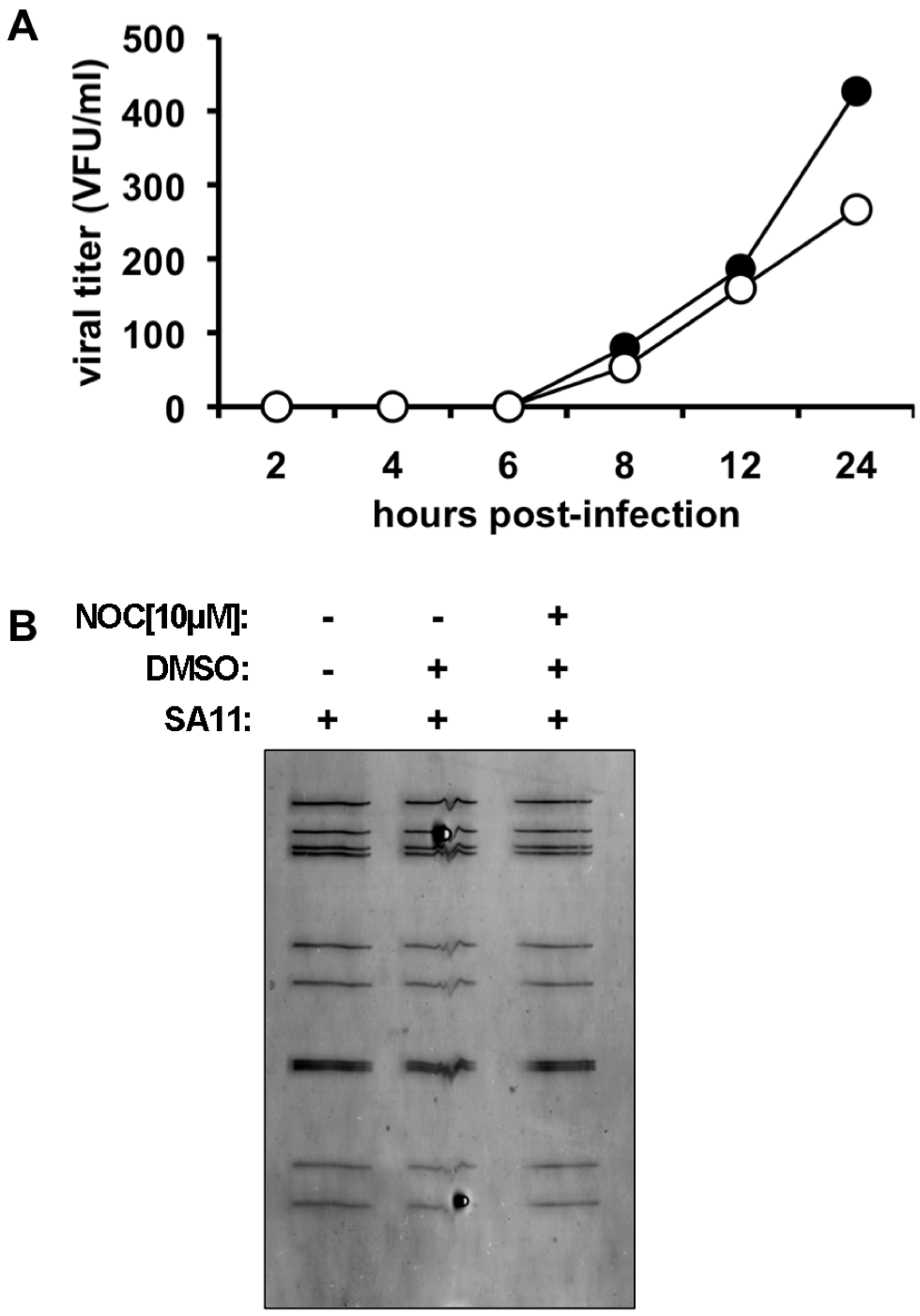

Supplement: Figure S5 — Rotavirus replication is not affected by nocodazole treatment. (A) Viral fitness curve of simian rotavirus SA11-infection in treated and non-treated CV-1 cells [MOI; 25 VFU/cell]. At 1 hpi, cells were untreated ( ) (2% DMSO) or treated with 10 µM nocodazole ( and cells were harvested at 2, 4, 6, 8, 12 and 24 hpi. The viral titers (VFU/ml) for each time point were determined using NSP5-EGFP/MA104 and were plotted. (B) SA11-infected CV-1 cells [MOI; 25 VFU/cell] were treated at 1 hpi, with 10 µM nocodazole. At 18 hpi, cells were harvested, virus genome was extracted, resolved in a 10% SDS-PAGE and silver stained to detect dsRNA genome segments. (TIF) [file pone.0047947.s005.tif]

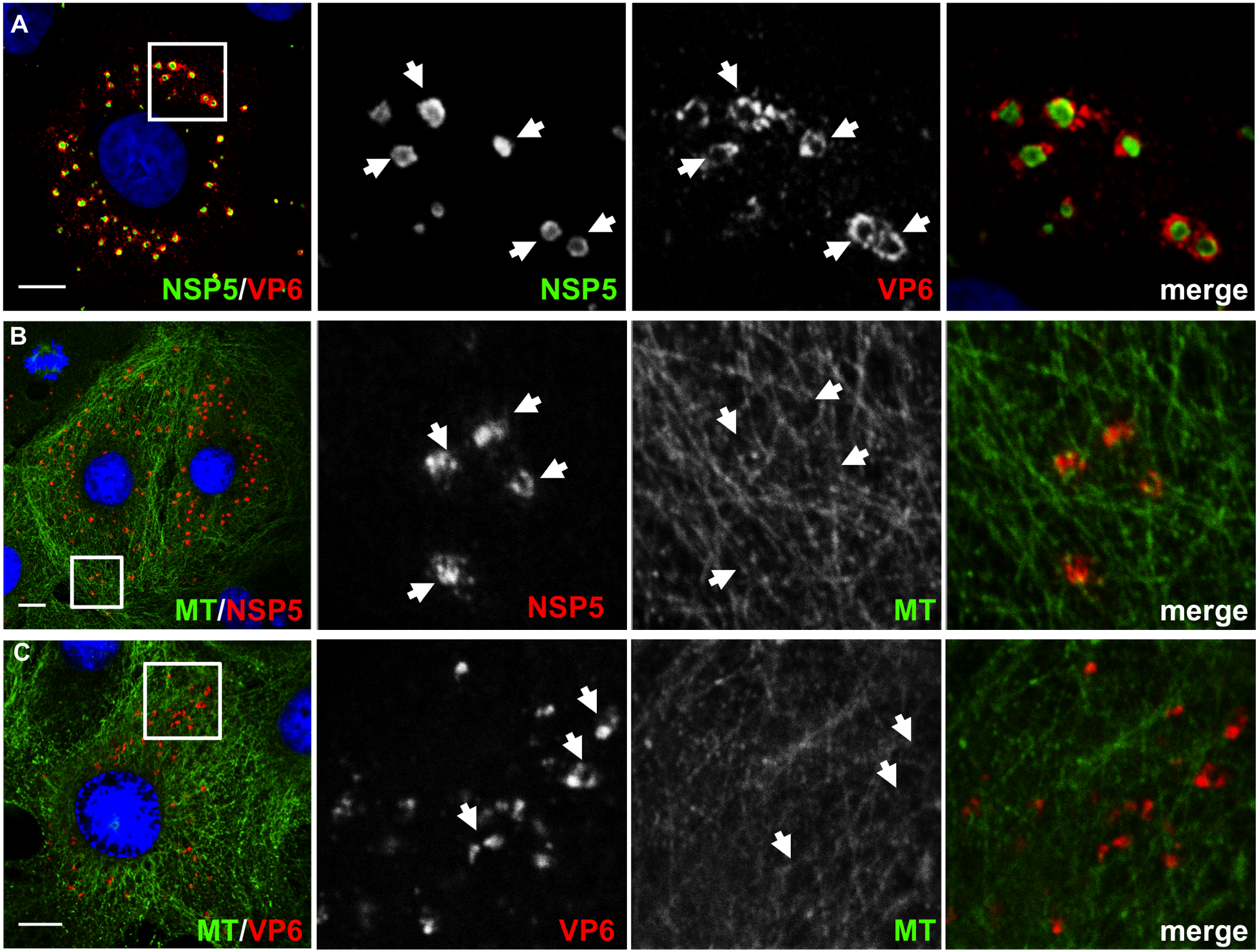

Supplement: Figure S6 — Immunofluorescence analysis of viroplasms in SA11-infected CV-1 cells [MOI; 25 VFU/cell] at 6 hpi. (A) The viroplams internal and external (white arrows) domains were detected with specific anti-NSP5 serum (green) and with mAb anti-VP6 antibody (red), respectively. (B) Co-immunofluorescence analysis of SA11 viroplasms stained with a specific anti-NSP5 serum (red) and MTs stained with mAb anti-tubulin conjugated to Atto 488 (green). (C) Co-immunofluorescence analysis of SA11 viroplasms stained with mAb anti-VP6 (red) and MTs stained with mAb anti-tubulin conjugated to Atto 488 (green). The white-boxed area shows the site for picture enlargement in which MT-network (white arrows) co-localize with viroplasms. Scale bars are 10 µm. (TIF) [file pone.0047947.s006.tif]

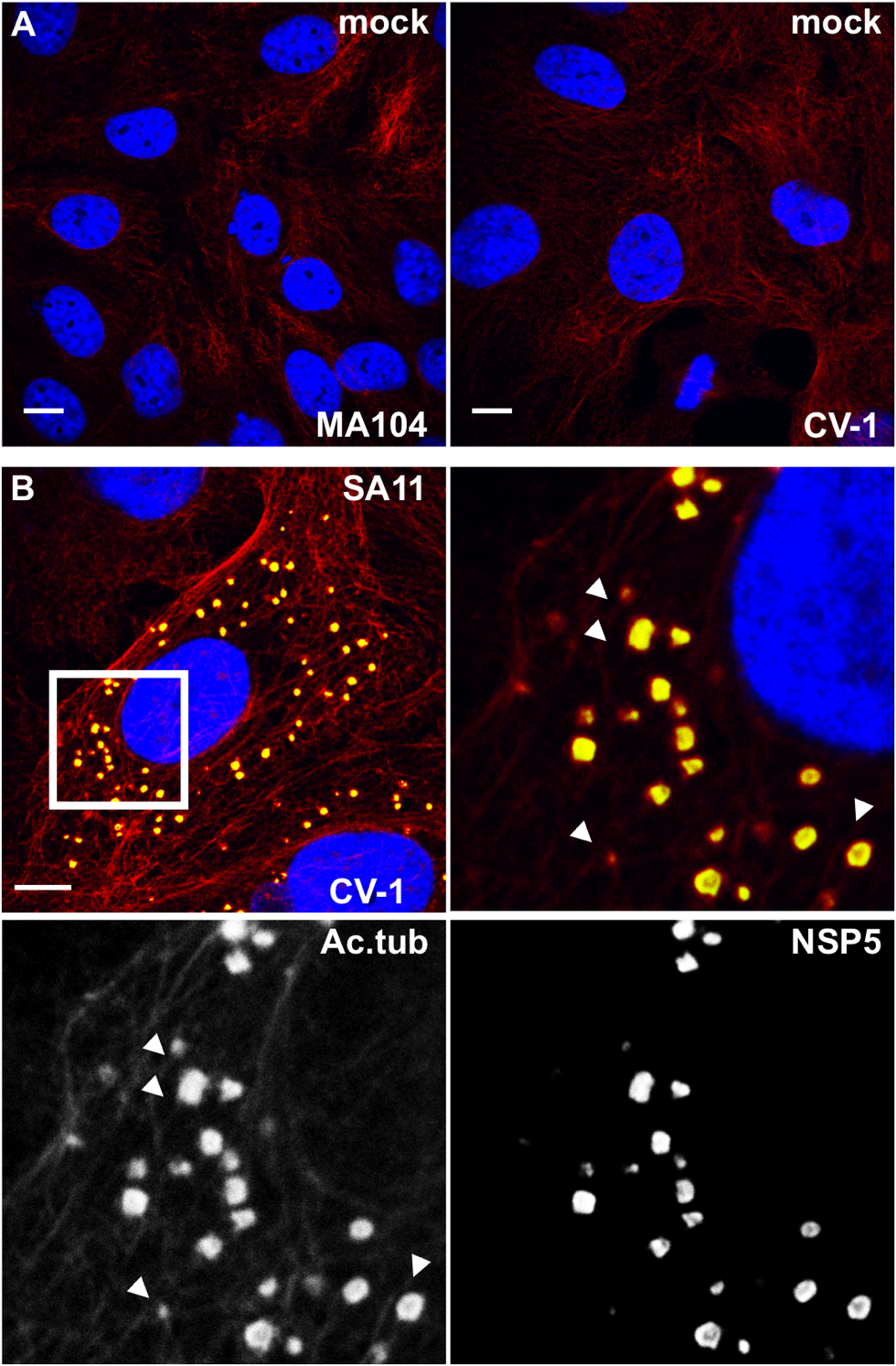

Supplement: Figure S7 — Acetylated MTs in SA11-infected CV-1 cells. (A) Immunostaining for acetylated MTs (mAb anti- acetylated alpha-tubulin, red) of MA104 (left) and CV-1 (right) cells. (B) Immunofluorescence analysis of SA11-infected CV-1 cells at 6 hpi, showing viroplasms (anti-NSP5, green), acetylated tubulin (mAb anti-acetylated tubulin, red) and nuclei (DAPI, blue), upper image left. The white-boxed area shows an enlarged image that indicates the localization of the hyper-acetylated MTs (white arrowheads) in the viroplasm region. Scale bars are 10 µm. (TIF) [file pone.0047947.s007.tif]

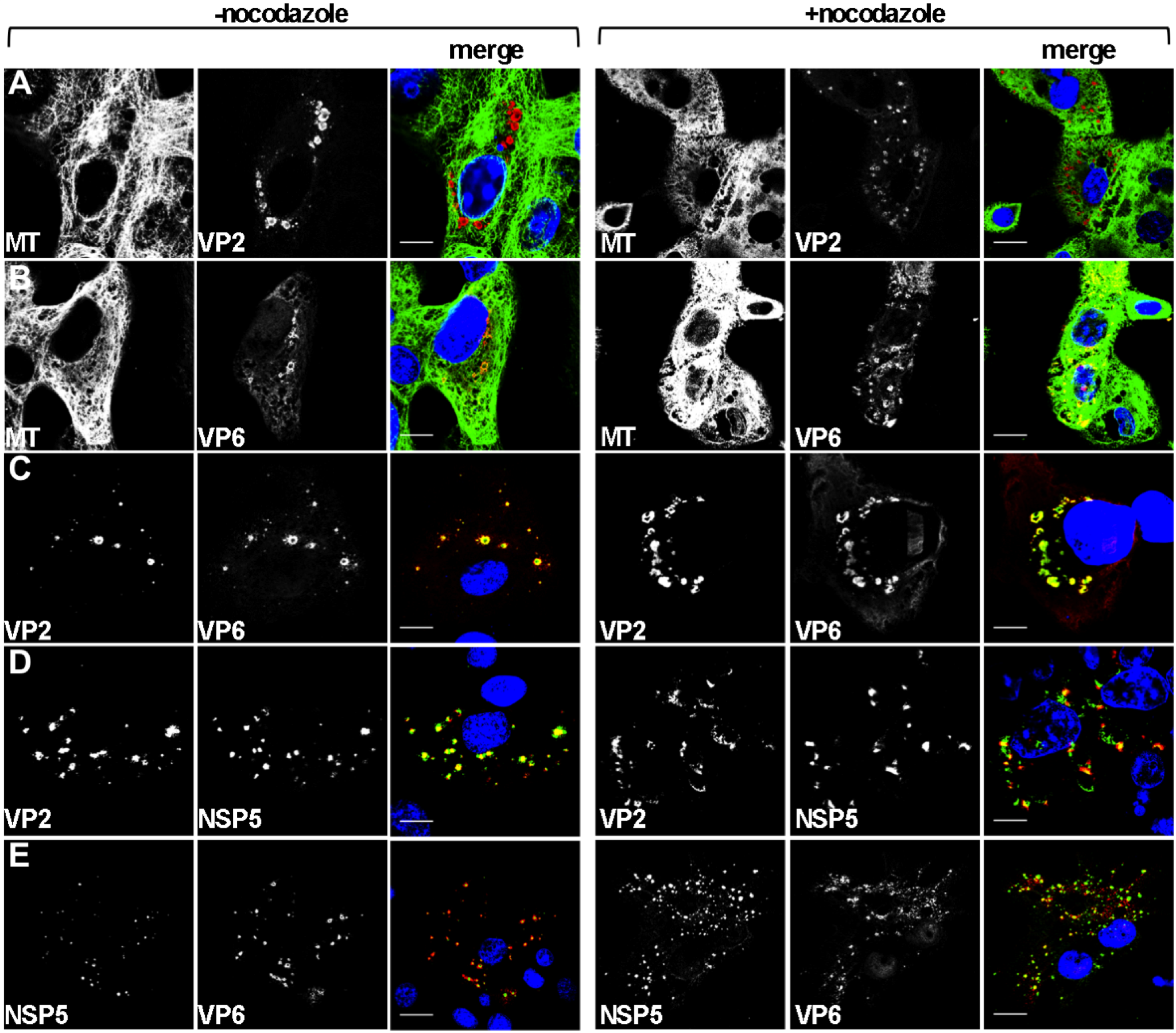

Supplement: Figure S8 — Viroplasms show a disperse morphology upon nocodazole treatment. Rotavirus SA11-infected CV-1 cells [MOI; 25 VFU/cell] were untreated (left panel) or treated with 10 µM nocodazole (right panel) for 5 hours before fixation. At 6hpi, cells were fixed with methanol and co-immunostained for: (A) MT (mAb anti-alpha tubulin conjugated to atto 488, green) and VP2 (anti-VP2, red); (B) MT (mAb anti-alpha tubulin conjugated to atto 488, green) and VP6 (mAb anti-VP6, red); (C) VP2 (anti-VP2, red) and VP6 (mAb anti-VP6, green); (D) VP2 (anti-VP2, green) and NSP5 (anti-NSP5, red) and (E) NSP5 (anti-NSP5, green) and VP6 (mAb anti-VP6, red). In each panel, the left and middle columns show the separate acquisitions and the right column shows the merged images. Nuclei are stained with DAPI (blue). Scale bars are 10 µm. (TIF) (TIF) [file pone.0047947.s008.tif]
