# Supplementary material for: Rotavirus Viroplasm Fusion and Perinuclear Localization Are Dynamic Processes Requiring Stabilized Microtubules
Source: PLoS One. 2012 Oct 23;7(10):e47947. doi: 10.1371/journal.pone.0047947 (PMC3479128; doi:10.1371/journal.pone.0047947)
Supplement: Table S1 — Primers for plasmid pHSV-1-NSP2-mCherry construction. (DOCX) [file pone.0047947.s009.docx]

**Table S1**. Primers for plasmid pHSV-1-NSP2-mCherry construction.

| **amplified DNA segment** | **primers** | **oligonucleotide sequence** |
| --- | --- | --- |
| NSP2 | forward | 5’-gatcgaattc**atg**gctgagctagcttgcttt-3’ |
|  | reverse | 5’-gatcacgcgtaacgccaacttgagaaacttc-3’ |
| mCherry | forward | 5’-gatcgtcgac**atg**gtgagcaagggcgaggag-3’ |
|  | reverse | 5’-gcatcccgggcttgtacagctcgtccatgcc-3’ |
| pCI-NSP2-mCherry | forward | 5’-gatcgtcgac**atg**gctgagctagcttgcttt-3’ |
|  | reverse | 5’-gatcgaattc**tta**cttgtacagctcgtccat-3’ |

^*^Restriction enzyme sites are underlined

^**^Initiation and stop codons are labeled in bold
